# *Berberis turcomanica* berries: an integrated evaluation of antioxidant, enzyme inhibitory, and antimicrobial activities, phytochemical profile and *in silico* analysis

**DOI:** 10.1039/d6ra02922a

**Published:** 2026-06-05

**Authors:** Serdar Korpayev, Emre Can Buluz, Jasmina Glamočlija, Neda Popović, Uroš Gašić, Dejan Stojković, Hemra Hamrayev, Mirap Agamuradov, Savaş Kaya, Enver Saka, Gökhan Zengin

**Affiliations:** a Biotechnology Institute, Ankara University 06100 Ankara Turkey; b Institute of Science, Department of Physics, Ege University İzmir 35100 Turkey; c Department of Plant Physiology, Institute for Biological Research “Siniša Stanković” - National Institute of Republic of Serbia, University of Belgrade Bulevar Despota Stefana 142 11000 Belgrade Serbia; d Department of Chemical and Petroleum Engineering, University of Calgary, 2500 University Drive NW Calgary Alberta T2N 1N4 Canada; e Saint Petersburg State Pediatric Medical University Russian Federation; f Faculty of Science, Department of Chemistry, Sivas Cumhuriyet University Sivas 58140 Turkey; g Department of Biology, Science Faculty, Selcuk University Konya Turkey gokhanzengin@selcuk.edu.tr

## Abstract

*Berberis turcomanica*, commonly known as the Turcoman Barberry, is a lesser-studied deciduous shrub of the Berberidaceae family, primarily distributed in Central Asia with its heartland in Turkmenistan. The extracts of the berries of *Berberis turcomanica* (BTB) were subjected to *in vitro* assays to assess antioxidant capacity (DPPH, ABTS, FRAP, CUPRAC), enzyme inhibitory activities (acetylcholinesterase, butyrylcholinesterase, tyrosinase, α-amylase, and α-glucosidase), antimicrobial potential (against selected bacterial and fungal strains), and cytotoxic effects on human HaCaT cell lines. Among the 28 chemical constituents analyzed using UHPLC-DAD-QqQ-MS/MS. The extracts exhibited strong antioxidant activity, supported by high total phenolic and flavonoid contents. Significant enzyme inhibition, particularly against tyrosinase and α-glucosidase, suggests potential applications in managing hyperpigmentation and diabetes. Both fruit extracts also exhibited promising antibacterial effects against several bacterial strains and moderate antifungal activity; however, the extracts demonstrated low cytotoxicity toward non-cancerous human keratinocyte HaCaT cells, with IC_50_ values exceeding 400 µg mL^−1^, indicating a favorable safety profile and good biocompatibility under the tested conditions. Molecular docking, MD simulation-based analyses, and DFT calculations provided supportive insights into the potential activities of selected individual compounds identified in the extracts, partially complementing the experimentally obtained findings. These results present the first detailed pharmacological and chemical investigation of *B. turcmanica* berries in two extraction methods and support their potential use as a multifunctional natural agent in pharmaceutical and nutraceutical applications.

## Abstract 2

These findings constitute the first comprehensive pharmacological and chemical study of *B. turcmanica* berries and highlight their promise as a versatile natural agent for both pharmaceutical and nutraceutical applications.

## Graphical abstract

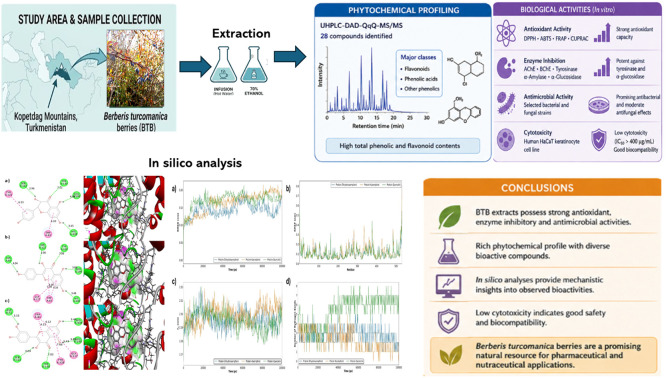

## 1 Introduction

The prickly deciduous shrub *Berberis turcomanica*, a member of the Berberidaceae family, is indigenous to Central Asia and can be found naturally in Turkmenistan, Iran, and Afghanistan.^1,2^ With its bright yellow blooms and tiny crimson or black berries, this plant, which can grow up to 2 meters tall, has long been used in traditional medicine and cooking.^3^ For generations, *B. turcmanica* has been an essential part of Central Asian ethnobotanical communities.^3^ Because of its rich phytochemical profile and wide range of ethnobotanical uses, *B. turcmanica* has substantial cultural and scientific importance beyond its natural habitat.^3^

Previous phytochemical studies have reported the presence of rare alkaloids such as turconidine and turcomanidine in *Berberis turcomanica*.^4,5^ However, the available literature is limited and mainly focused on the isolation and characterization of individual alkaloids rather than comprehensive phytochemical profiling. Notably, *Berberis turcomanica* serves as the main natural source of the rare alkaloid Turconidine and Turcomanidine, as first reported in the study *Berberis Alkaloids* XXXI and XXXVI.^5,6^ Numerous bioactive substances, such as alkaloids, flavonoids, phenolics, and tannins, have been found in *B. turcmanica* by phytochemical investigations.^7^ The plant is becoming more and more popular in pharmacological study because of these components, which support its claimed anti-inflammatory, anti-cancer, antioxidant, and antibacterial qualities.^8–11^ Similar biological activities have also been widely reported in other *Berberis* species, particularly due to the presence of berberine, phenolic acids, and flavonoids.^8,12^

A mainstay of regional cooking, the plant's tart, sour berries are used to make Sharbat, a cool, sweet-and-sour drink, and to flavor foods like salads, stews, and soups. In addition to its functional applications, *B. turcmanica* is used as an ornamental plant in landscaping because of its eye-catching flowers and berries. Its root bark, which is abundant in the yellow alkaloid berberine, has long been used as a natural dye for yarns and textiles.^3,11,13^

The biological potential of *Berberis turcomanica'*s berries collected from the natural habitats of Turkmenistan has not received enough scientific attention, despite the fact that a large portion of the literature on the plant has concentrated on its roots and alkaloid-rich sections, especially since Turcomanidine and Berberine^14^ were discovered. Moreover, most previous studies on *Berberis* species have focused primarily on roots, stems, or purified alkaloids, while comparatively limited information is available regarding the phytochemical composition and biological activities of berry extracts, particularly from *B. turcomanica*.^8,12^ Although several *Berberis* species such as *B. vulgaris*, *B. aristata*, and *B. integerrima* have been extensively investigated for their phytochemical composition and biological activities, limited information is currently available regarding the berry extracts of *Berberis turcomanica berries*, particularly in terms of their comparative phytochemical profile, antioxidant capacity, enzyme inhibitory potential, and antimicrobial activities.^8,12^ Therefore, the present study aimed to evaluate whether *B. turcomanica* berry extracts exhibit distinct biological and phytochemical characteristics that may support their potential use as natural functional or pharmacological agents. In light of their rich pigmentation and historical use, the current study examines the phytochemical makeup and anti-oxidant and enzyme inhibitory bioactivities of *B. turcmanica* berry extracts for the first time in the literature. The plant has the potential to be used as a natural therapeutic agent because infusion and 70% extracts have shown effectiveness in preventing the growth of bacteria and fungi, including some strains that are resistant to antibiotics. In addition, evaluating both infusion and hydroalcoholic extracts may provide useful insight into the influence of extraction solvents on phytochemical composition and biological activity.^15,16^ Thus, the pharmacological potential of *B. turcmanica* berries is anticipated to be better understood thanks to this thorough approach, opening the door for its possible use in next pharmaceutical and nutraceutical applications.

## 2 Materials and methods

### 2.1 Extraction procedures for study samples

**Fig. 1:**
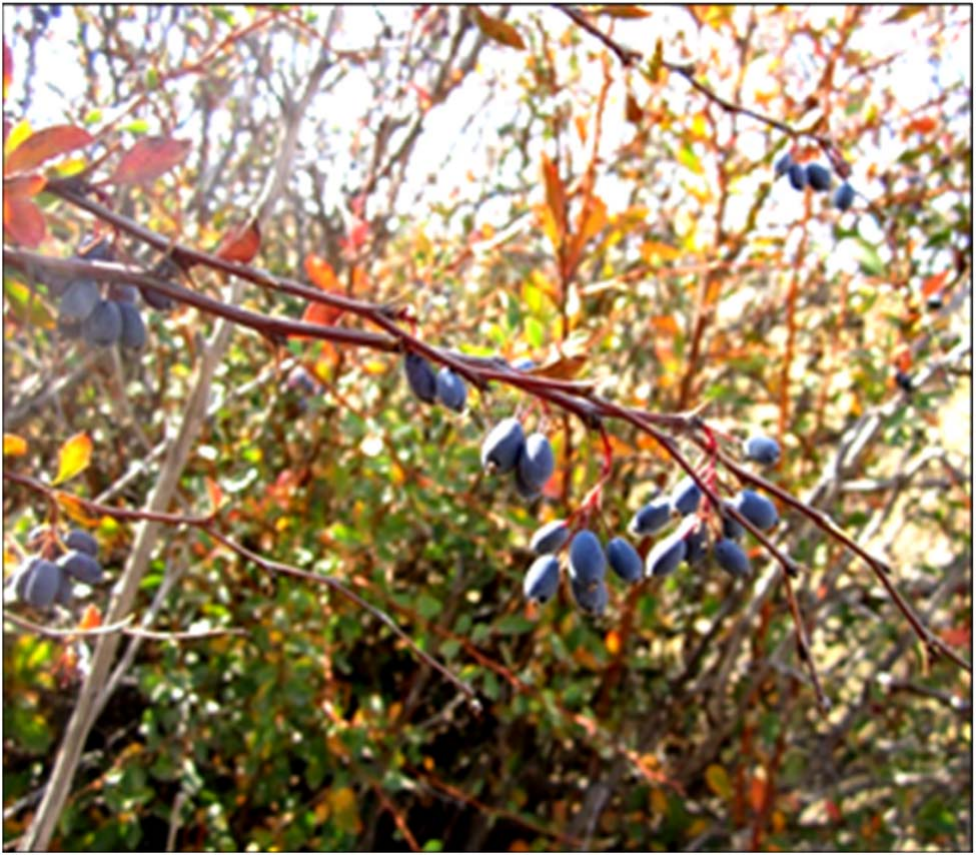
Images of *Berberis turcomanica* berries (BTB).

In 2022, *Berberis turcomanica* berries (BTB) were collected from the Kophetdag mountains, Akhal, Turkmenistan by biologist Batyr Danatarov (Fig. 1). The collected BTF material was cleaned, dried under sunlight for 7 days by spreading in a single layer on clean cloths and stored in bags until extraction. The BTB was covered with fine mesh to prevent contamination by insects and dust. The dried BTB samples were then cut into small pieces to increase the surface area. After drying, the samples were stored in airtight bags under dry and dark conditions until extraction in order to minimize possible phytochemical degradation during storage. The extraction of these materials was carried out using 70% ethanolic and infusion extraction methods, following the procedure used in our previous study.^17^

### 2.2 Phytochemical profiling of extracts using UHPLC-DAD-QqQ-MS/MS

ThermoFisher UHPLC-MS analysis was carried out at 40 °C utilizing a Syncronis C18 column (100 × 2.1 mm, 1.7 µm) in conjunction with a triple quadrupole MS. Information about the MS parameters and chromatographic conditions is based on what Radović *et al.*^18^ described. At 254 and 280 nm, detection was performed using a 5 µL injection volume. Xcalibur v2.2 software was used for data processing and collecting. Results were reported in mg kg^−1^ after phenolic components were discovered and measured in comparison to reliable standards.^19^

Validation parameters (retention times, precursor and product ions with specified collision energies, calibration range, equation parameters, determination coefficient (*R*^2^), LOD (limit of detection), and LOQ (limit of quantification) of used UHPLC-MS/MS method are given in the Table S1. LOD (limit of detection) and LOQ (limit of quantitation) are calculated in excel using a calibration curve (linear regression) based on the residual standard deviation (*σ*) and slope (*S*) of the curve. The formulas are: LOD = (3 × *σ*)/*S* and LOQ = (10 × *σ*)/*S*. Very good linearity was shown for each compound with a coefficient of determination (*R*^2^) over 0.99.

### 2.3 Biological activity evaluation of *Berberis turcomanica* berry extracts

Total Phenolic (TPC) and Flavonoid Content (TFC) analysis, antioxidant, enzyme inhibitory, antimicrobial, and cytotoxic activities of *Berberis turcomanica* berry extracts were evaluated by following our previously published article.^17,20^ Antibacterial and antifungal effects were assessed using the modified microdilution method. The bacterial strains tested included *Staphylococcus aureus*, *Bacillus cereus*, *Micrococcus luteus*, *Listeria monocytogenes*, *Pseudomonas aeruginosa*, *Salmonella Typhimurium*, *Escherichia coli*, and *Enterobacter cloacae*. Antifungal activity was evaluated against *Aspergillus fumigatus*, *A. versicolor*, *A. ochraceus*, *A. niger*, *Trichoderma viride*, *Penicillium funiculosum*, *P. ochrochloron*, and *P. verrucosum* var*. Cyclopium*.^20^ All experimental details are given in the supplemental materials.

### 2.4 Density functional theory (DFT) calculations

Through Conceptual Density Functional Theory (CDFT) based structural parameters and electronic structure principles are frequently preferred by computational chemists in the analysis of global and local reactivity of chemical systems and in the prediction of biological activity. After experimentally elucidating the phytochemical profile of the studied extracts, this section presents DFT calculations to reliably describe the structure–reactivity relationships of the major components in the extracts. Here, we used Orca software^21^ and computations were performed *via* B3LYP/6-31+G(d,p) calculation level. Conceptual Density Functional Theory (CDFT) defines the chemical reactivity descriptors such as chemical potential (*µ*), electronegativity (*χ*), chemical hardness (*η*) and softness (*σ*) as:^22,23^
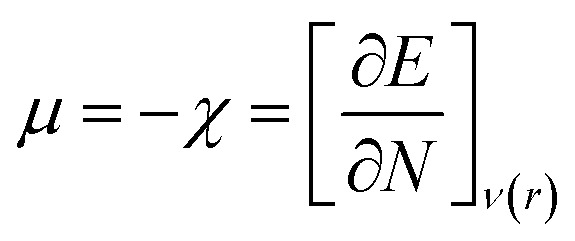

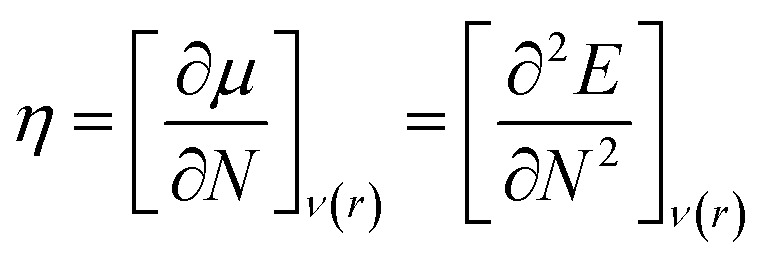
*σ* = 1/*η*With the application of the finite differences approach to afore given mathematical relations including the total electronic energy (*E*) and total number of the electrons (*N*), one can obtain the following equations based on the ionization energy (*I*) and electron affinity (*A*) of the chemical systems
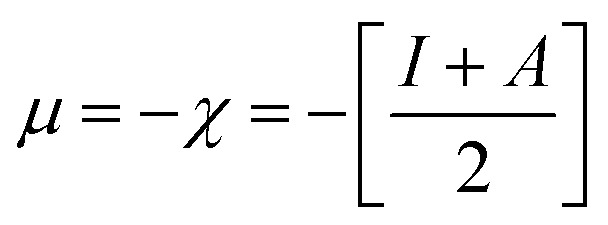
*η* = *I* − *A**σ* = 1/[*I* − *A*]

Electrophilicity index (*ω*) is among the widely preferred parameters to explain the reactivity of the molecular systems. This concept has important applications, particularly in elucidating the mechanisms of organic reactions. To predict the first and second electrophilicity indexes, the following equations below have been derived. These electrophilicity indexes are based on ground state parabola and valence state parabola models of CDFT, respectively.^24,25^*ω*_1_ = *χ*^2^/2*η* = *µ*^2^/2*η*
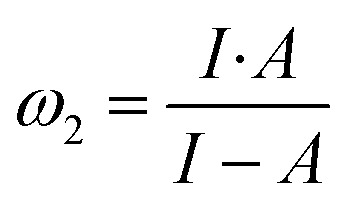


It is important to note that Koopmans Theorem^26^ was used to estimate the ionization energies and electron affinities of the dominant components of the extract under study.

### 2.5 Molecular docking

Following DFT-based geometry optimization, the energy-minimized conformations of the selected twelve molecules were subjected to molecular docking analysis using the PLANTS software.^27^ The three-dimensional structures of the enzymes investigated in the experimental studies—acetylcholinesterase (AChE, PDB ID: 1QTI^28^), butyrylcholinesterase (BChE, PDB ID: 1XLW^29^), tyrosinase (TYR, PDB ID: 1WX2 (ref. 30)), amylase (AML, PDB ID: 9FZ3 (ref. 31)), and glucosidase (GLC, PDB ID: 2RGL) (ref. 32)—were obtained from the RCSB Protein Data Bank.^33^ The binding site was defined according to the locations of the co-crystallized ligands in the crystal structures of the target enzymes. Re-dock analysis was performed on each protein ligand molecule with its crystal structure, and RMSD values were obtained. Arbutin was used as a control molecule for the TYR enzyme. This approach was employed to demonstrate the docking protocol's capability to generate novel ligand binding poses. Protein preparation was performed using the Dock Prep module in UCSF Chimera, which included the removal of non-standard residues and water molecules, the addition of hydrogen atoms under physiological protonation conditions (pH 7.0), and the assignment of partial charges. The molecular structures optimized in the preceding DFT step were converted into appropriate formats (.mol2) and used as inputs for the docking studies. For each molecule, a single binding pose was selected based on the PLANTS scoring function. The resulting protein-ligand complexes were visualized in PyMOL^34^ and further analyzed using BIOVIA Discovery Studio Visualizer.^35^

### 2.6 Molecular dynamics simulations and free energy calculations

Molecular dynamics (MD) simulations were carried out using GROMACS^33^ to examine the dynamic behavior and structural stability of the protein-ligand complexes derived from molecular docking, where for each target enzyme the three ligands with the most favorable docking scores were selected and subjected to MD simulations to ensure a consistent and comparative evaluation of the top-ranked binding poses across all systems. Protein parameters were assigned using the AMBER ff99SB force field,^36^ while ligand topologies and force field parameters were generated with the ACPYPE tool.^37^ Each system was embedded in a dodecahedral simulation box with a minimum solute-to-boundary distance of 1.0 nm and solvated with TIP3P water molecules. System charge neutrality was achieved by adding appropriate counterions. Prior to production runs, energy minimization was performed using the steepest descent algorithm to remove unfavorable steric interactions. The minimized systems were subsequently equilibrated in two stages: a 250 ps constant-volume (NVT) phase followed by a 250 ps constant-pressure (NPT) phase, with positional restraints applied to heavy atoms. Temperature was maintained at 300 K using the V-rescale thermostat, while pressure was controlled at 1 bar with the Parrinello–Rahman barostat. Long-range electrostatic interactions were handled using the Particle Mesh Ewald (PME) method, with a cutoff of 1.4 nm applied to both electrostatic and van der Waals interactions. After the equilibration steps, 100 ns production MD simulations were carried out for each protein-ligand complex. Trajectories were analyzed using GROMACS to evaluate structural stability and flexibility, including root-mean-square deviation (RMSD), root-mean-square fluctuation (RMSF), radius of gyration (*R*_g_) and Hydrogen bond analysis. Binding free energy calculations were performed using the gmx_MMPBSA^38^ tool on 500 frames of the MD trajectory with a generalized Born model (igb = 5) and an ionic strength of 0.150 M. Binding free energies were estimated by averaging over snapshots extracted from the entire MD trajectory to ensure adequate conformational sampling. In addition, energy decomposition analyses were performed to elucidate the individual contributions of van der Waals interactions, electrostatic interactions, polar solvation energy, and non-polar solvation energy to the overall binding affinity.

### 2.7 Statistical analyses

The mean ± SD of three separate experiments is used to show the data. Statistical analysis was conducted using Welch's *t*-test, and differences were considered statistically significant at *p* < 0.05.

## 3 Results and discussion

### 3.1 Total phenolic and flavonoid content analysis of BTB

Table 1 shows the total phenolic content (TPC) and total flavonoid content (TFC) of two different extracts (infusion and 70% ethanolic) from BFT. For the infusion extract of BFT, the TPC is 82.99 ± 0.58 mg GAE per g, while the TFC is 5.27 ± 0.21 mg RE per g. For the 70% ethanolic extract of *Berberis turcomanica*, the TPC is 4.70 ± 0.22 mg GAE per g, while the TFC is 0.47 ± 0.044 mg RE per g, aligning with previous findings in other *Berberis* species such as *B. vulgaris* and *B. İntegerrima*.^7,8,39^ Statistical analysis revealed that the differences in both TPC and TFC values between the infusion and 70% ethanolic extracts were statistically significant (*p* < 0.05). The markedly higher TPC and TFC values observed in the infusion extract may be associated with the higher extraction efficiency of polar phenolic acids and related hydrophilic compounds during hot water extraction. In contrast, the 70% ethanolic extract may have preferentially extracted less polar flavonoid compounds, suggesting selective solvent-dependent phytochemical recovery rather than experimental inconsistency.^40^ This interpretation was further supported by the UHPLC-DAD-QqQ-MS/MS results discussed below, where the infusion extract showed substantially higher concentrations of caffeoylquinic acid and caffeic acid derivatives compared to the 70% ethanolic extract.

**Table 1:** Total phenolic (TPC) and flavonoid (TFC) content in the tested extracts^*^^a^

Extracts	TPC (mg GAE per g)	TFC (mg RE per g)
Infusion	82.99 ± 0.58^a^	5.27 ± 0.21^a^
70% Ethanolic	4.70 ± 0.22^b^	0.47 ± 0.044^b^

a Values are reported as mean ± SD of three parallel measurements. GAE: gallic acid equivalent; RE: rutin equivalent. Different superscript letters within the same row indicate statistically significant differences between extracts (*p* < 0.05) UHPLC-DAD-QqQ-MS/MS.

Table 2 details the concentrations (mg kg^−1^) of phenolic and flavonoid compounds in aqueous and 70% ethanolic extracts of a BTB, revealing distinct extraction efficiencies. The aqueous extract was notably rich in 5-*O*-caffeoylquinic acid (1927.18 mg kg^−1^), followed by caffeic acid (198.04 mg kg^−1^), 3-*O*-caffeoylquinic acid (132.47 mg kg^−1^), rutin (135.49 mg kg^−1^), and *p*-coumaric acid (132.41 mg kg^−1^). In contrast, the 70% ethanolic extract contained lower levels of 5-*O*-caffeoylquinic acid (1477.48 mg kg^−1^) and caffeic acid (97.49 mg kg^−1^) but higher concentrations of quercetin 3-*O*-rhamnoside (298.87 mg kg^−1^), quercetin (116.61 mg kg^−1^), and isorhamnetin (108.86 mg kg^−1^). Statistical analysis demonstrated significant differences (*p* < 0.05) between the extracts for most quantified compounds, whereas rutin showed no statistically significant difference between the two extraction methods. Several compounds, including isoorientin, catechin gallate, ellagic acid, naringin, chrysin, and pinocembrin, were not detected (NF) in either extract, suggesting their absence or presence below detection limits.^17^ Specifically, the absence of these compounds may indicate that they are either not naturally present in *Berberis turcomanica* berries or occur at concentrations below the detection limits of the analytical method used. In addition, differences in solvent polarity and extraction efficiency may also have influenced the recovery of certain phytochemicals. The 70% ethanolic extract uniquely contained compounds such as isorhamnetin 3-*O*-glucoside (71.57 mg kg^−1^), myricetin (7.85 mg kg^−1^), eriodictyol (3.29 mg kg^−1^), luteolin (5.13 mg kg^−1^), apigenin (7.35 mg kg^−1^), and hispidulin (0.87 mg kg^−1^), which were absent in the infusion extract, indicating a greater affinity for flavonoids in ethanol-based extraction. This observation may be associated with the intermediate polarity of aqueous ethanol, which can improve the solubility and extraction efficiency of less polar flavonoid aglycones and glycosides.^15,16^

**Table 2:** Profiling of bioactive compounds in the tested extracts extracts (mg kg^−1^)^a^

Compounds	Infusion	70% Ethanolic extract
3-*O*-Caffeoylquinic acid	132.47 ± 1.26^a^	54.81 ± 1.43^b^
5-*O*-Caffeoylquinic acid	1927.18 ± 12.86^a^	1477.48 ± 36.88^b^
Caffeic acid	198.04 ± 11.71^a^	97.49 ± 2.69^b^
Rutin	135.49 ± 1.82^a^	132.18 ± 12.48^a^
Vitexin	0.37 ± 0.02^a^	0.20 ± 0.01^b^
*p*-Coumaric acid	132.41 ± 1.00^a^	66.87 ± 0.75^b^
Quercetin 3-*O*-glucoside	88.30 ± 2.53^a^	130.06 ± 6.35^b^
Isorhamnetin 3-*O*-rutinoside	105.77 ± 9.12^a^	91.03 ± 8.82^b^
Isorhamnetin 3-*O*-glucoside	NF	71.57 ± 1.86
Quercetin 3-*O*-rhamnoside	228.08 ± 2.30^a^	298.87 ± 0.67^b^
Kaempferol 3-*O*-glucoside	46.65 ± 2.47^a^	73.05 ± 1.29^b^
Myricetin	NF	7.85 ± 0.17
Dihydrokaempferol	7.70 ± 0.24^a^	20.96 ± 0.25^b^
Eriodictyol	NF	3.29 ± 0.22
Luteolin	NF	5.13 ± 0.40
Quercetin	29.21 ± 1.84^a^	116.61 ± 4.53^b^
Naringenin	0.52 ± 0.01^a^	1.67 ± 0.03^b^
Apigenin	NF	7.35 ± 0.13
Kaempferol	10.03 ± 0.21^a^	58.74 ± 1.88^b^
Hispidulin	NF	0.87 ± 0.01
Isorhamnetin	39.69 ± 1.76^a^	108.86 ± 3.80^b^

a Values are expressed as mean ± standard deviation of replicate analyses. Different superscript letters within the same row indicate statistically significant differences between extracts (*p* < 0.05). NF: not found.

Comparatively, the infusion extract favored phenolic acids (*e.g.*, caffeoylquinic acids, *p*-coumaric acid), while the 70% ethanolic extract excelled in extracting flavonoids (*e.g.*, quercetin, kaempferol, isorhamnetin). To illustrate, quercetin and kaempferol levels increased from 29.21 mg kg^−1^ and 10.03 mg kg^−1^ in the aqueous extract to 116.61 mg kg^−1^ and 58.74 mg kg^−1^ in the 70% ethanolic extract, respectively. This suggests that solvent polarity significantly influences the phytochemical profile, with 70% ethanolic extract enhancing flavonoid solubility and aqueous extraction prioritizing polar phenolic acids. The higher abundance of flavonoids in the ethanolic extract may contribute to enhanced antioxidant and pharmacological activities, as flavonoids are widely recognized for their radical scavenging and anti-inflammatory properties.^41,42^ These differences may impact the extracts' bioactivity, warranting further investigation into their therapeutic potential. The findings show that BTB may serve as a valuable source of natural antioxidants, potentially useful in functional foods or nutraceutical applications. Moreover, the presence of high phenolic and flavonoid concentrations supports the traditional medicinal use of *Berberis* fruits in treating inflammation and microbial infections.^8,43^

### 3.2 Antioxidant properties

The antioxidant activity of *Berberis turcomanica* berries infusion and 70% ethanolic extracts was evaluated using six assays: PBD, DPPH, ABTS, CUPRAC, FRAP, and MCA. Activity was expressed as mmol TE per g or mg TE per g for PBD, DPPH, ABTS, CUPRAC, and FRAP, and mg EDTAE per g for MCA (Table 3). The infusion extracts consistently outperformed the 70% ethanolic extract across all assays. For PBD, the infusion extract recorded 1.83 mmol TE per g, compared to 0.54 mmol TE per g for the 70% ethanolic extract. Statistical analysis confirmed that the difference between the extracts in the PBD assay was significant (*p* < 0.05). In DPPH and ABTS assays, the infusion extract yielded 135.02 mg TE per g and 262.86 mg TE per g, respectively, while 70% ethanolic extract values were unreported. CUPRAC and FRAP assays further highlighted this disparity, with the infusion extract at 332.73 mg TE per g and 177.72 mg TE per g, respectively, *versus* 18.85 mg TE per g and 9.71 mg TE per g for the 70% ethanolic extract. These differences were also statistically significant (*p* < 0.05), indicating markedly higher reducing and radical scavenging capacities of the infusion extract. The use of multiple antioxidant assays enabled the evaluation of different antioxidant mechanisms, including radical scavenging, reducing power, and metal chelation capacity. The stronger performance of the infusion extract in PBD, CUPRAC, and FRAP assays suggests a greater electron-donating and reducing ability, whereas the comparable MCA values indicate that both extracts possess similar metal chelating properties.^17^ These findings demonstrate that the antioxidant behavior of the extracts may vary depending on the reaction mechanism evaluated in each assay. In the MCA assay, the infusion extract (1.51 mg EDTAE per g) slightly exceeded the 70% ethanolic extract (1.38 mg EDTAE per g), though the difference was minimal. No statistically significant difference was observed between the extracts in the MCA assay (*p* > 0.05). However, direct comparison among antioxidant assays should be interpreted cautiously because each method is based on different reaction mechanisms and expressed in different units reflecting distinct antioxidant properties. The high quantity of flavonoids and phenolic acids, which are known to neutralize free radicals through hydrogen atom or electron donation processes, is probably what causes this potent antioxidant activity.^44^ The higher antioxidant activity observed in the infusion extract may be associated with its elevated levels of caffeoylquinic acid and caffeic acid derivatives identified in the phytochemical profiling analysis, suggesting a potential contribution of these phenolic compounds to radical scavenging capacity. The stronger antioxidant performance of the infusion extract may be associated with its higher phenolic acid content, particularly caffeoylquinic and caffeic acid derivatives, which are recognized as effective radical scavengers.^16,41^ Similar antioxidant-related findings have also been reported in other *Berberis* species. Ye *et al.* demonstrated that berberine-rich *Berberis vulgaris* extract exerted protective antioxidant effects against cholesterol overloading-induced apoptosis in primary mouse hepatocytes, further supporting the pharmacological relevance of *Berberis*-derived phytochemicals.^45^ Briefly, the infusion extract exhibited superior antioxidant activity, except in metal chelating capacity, where the two extracts were comparable, which is consistent with findings in related *Berberis* species such as *B. vulgaris* and *B. integerrima*.^2,7,44^ Previous studies on *Berberis* species have similarly reported strong antioxidant and enzyme inhibitory activities associated with high phenolic and flavonoid contents, supporting the relevance of these phytochemicals across the genus.^12,46^

**Table 3:** Antioxidant properties of the tested extracts^a^

Extracts	PBD (mmol TE per g)	DPPH (mg TE per g)	ABTS (mg TE per g)	CUPRAC (mg TE per g)	FRAP (mg TE per g)	MCA (mg EDTAE per g)
Infusion	1.83 ± 0.006^a^	135.02 ± 1.36	262.86 ± 1.43	332.73 ± 3.68^a^	177.72 ± 1.51^a^	1.51 ± 0.069^a^
70% Ethanol	0.54 ± 0.14^b^	na	na	18.85 ± 0.74^b^	9.71 ± 0.25^b^	1.38 ± 0.24^a^

a Values are reported as mean ± SD of three parallel measurements. TE: trolox equivalent; EDTAE: EDTA equivalent; na: not active. Different superscript letters within the same row indicate statistically significant differences between extracts (*p* < 0.05).

### 3.3 Enzyme inhibitory effects

The inhibitory effects of *Berberis turcomanica* infusion and 70% ethanolic extracts on AChE, BChE, α-amylase, α-glucosidase, and tyrosinase were assessed in Table 4, with results expressed in specific units (mg GALAE per g, mmol ACAE per g, or mg KAE per g). The infusion extract exhibited AChE inhibition at 2.97 mg GALAE per g (no BChE value reported), α-amylase at 0.05 mmol ACAE per g, α-glucosidase at 1.03 mmol ACAE per g, and tyrosinase at 15.74 mg KAE per g. The 70% ethanolic extract showed higher AChE inhibition (3.01 mg GALAE per g) and BChE inhibition (3.36 mg GALAE per g), with α-amylase at 0.25 mmol ACAE per g, α-glucosidase at 1.03 mmol ACAE per g, and tyrosinase at 66.69 mg KAE per g. Statistical analysis revealed no significant differences between the extracts for AChE and α-glucosidase inhibition (*p* > 0.05), whereas α-amylase and tyrosinase inhibitory activities differed significantly between the extracts (*p* < 0.05). These results are consistent with earlier research on other species of *Berberis*, particularly *B. vulgaris*, which showed notable inhibitory effects on α-glucosidase and cholinesterase because of its abundance of isoquinoline alkaloids like berberine.^8,47^ In general, the 70% ethanolic extract outperformed the infusion extract against AChE, BChE, and Tyrosinase, while the infusion extract showed slightly greater inhibition of α-Amylase; α-Glucosidase inhibition was equivalent between the two. The stronger tyrosinase inhibitory activity observed in the 70% ethanolic extract may be associated with its higher flavonoid content, as flavonoids such as quercetin and kaempferol derivatives are known to exhibit tyrosinase inhibitory potential.^48,49^ Interestingly, while the extracts showed strong antioxidant and enzyme inhibitory activities, they exhibited low cytotoxicity toward human HaCaT cell lines. Since HaCaT cells represent non-cancerous human keratinocytes commonly used for biosafety evaluation, the observed low cytotoxicity indicates good biocompatibility and suggests that the extracts may be considered safe for potential topical or biomedical applications.

**Table 4:** Enzyme inhibitory effects of the tested extracts^a^

Extracts	AChE (mg GALAE per g)	BChE (mg GALAE per g)	α-Amylase (mmol ACAE per g)	α-Glucosidase (mmol ACAE per g)	Tyrosinase (mg KAE per g)
Infusion	2.97 ± 0.0067^a^	na	0.05 ± 0.0016^a^	1.03 ± 0.01^a^	15.74 ± 0.29^a^
70% Ethanolic	3.01 ± 0.0046^a^	3.36 ± 0.015	0.25 ± 0.0089^b^	1.02 ± 0.01^a^	66.69 ± 0.46^b^

a Values are reported as mean ± SD of three parallel measurements. GALAE: galantamine equivalent; ACAE: acarbose equivalent; KAE: kojic acid equivalent; na: not active. Different superscript letters within the same row indicate statistically significant differences between extracts (*p* < 0.05).

### 3.4 Antibacterial activity

Table 5 summarizes the antibacterial activity of infusion and 70% ethanolic extracts of *Berberis turcomanica* berries against selected Gram-positive and Gram-negative bacterial strains. The 70% ethanolic extract consistently showed stronger antibacterial activity than the infusion, with MIC values ranging from 0.5 to 3.0 mg mL^−1^, compared to 1.0 to 3.0 mg mL^−1^ for the infusion. Notably, the 70% ethanolic extract was twice as potent against *E. coli* and *S. Typhimurium*, with MICs of 0.5 and 1.0 mg mL^−1^, respectively, *versus* 1.0 and 2.0 mg mL^−1^ for the infusion. MBC values mirrored this trend, with the 70% ethanolic extract requiring only 1.0–4.0 mg mL^−1^ to achieve bactericidal effects, while the infusion required up to 4.0 mg mL^−1^. The enhanced antibacterial activity of the 70% ethanolic extract may be associated with its higher content of flavonoids and other moderately polar phytochemicals identified in the compositional analysis, which may contribute synergistically to microbial growth inhibition.^50,51^ Both extracts were less potent than conventional antibiotics such as streptomycin (MIC 0.025–0.150 mg mL^−1^) and ampicillin (MIC 0.100–0.300 mg mL^−1^), however they demonstrated detectable broad-spectrum antibacterial activity against the tested strains. Nevertheless, comparisons between plant extracts and standard antibiotics should be interpreted cautiously because crude extracts contain complex mixtures of bioactive compounds rather than purified active substances. When compared to other *Berberis* species, the antibacterial profile of BTB is consistent with previous reports.^7,52^ Previous investigations on *Berberis vulgaris* and related species have similarly demonstrated antimicrobial activities associated with phenolic compounds and isoquinoline alkaloids, particularly berberine, supporting the antimicrobial relevance of phytochemicals across the genus.^14,53^ The *in vitro* study evaluating the effects of *Berberis vulgaris* ethanol extracts on bacteria linked to dental caries and different fungi primarily supports the MIC values and antimicrobial activity of the extracts. It reported antifungal MICs ranging from 70 to 90 µg mL^−1^ for *Aspergillus flavus* and related species, as well as MIC values of 64 µg mL^−1^ for *Streptococcus sobrinus* and 128 µg mL^−1^ for *Lactobacillus rhamnosus*.^54^ The observed antimicrobial effects may be associated with the presence of berberine and related isoquinoline alkaloids commonly reported within the genus. The 70% ethanolic extract's improved efficacy may reflect improved extraction efficiency of less polar bioactive constituents. This observation is also consistent with previous reports indicating that hydroalcoholic solvents improve the extraction efficiency of antimicrobial phytochemicals due to their intermediate polarity and ability to solubilize a broader range of bioactive constituents.^16^ Therefore, BTB, especially its 70% ethanolic form, may represent a source of bioactive compounds warranting further phytochemical and biological investigation, although its antimicrobial potency remains lower than conventional antibiotics and some previously reported *Berberis* extracts.^54^

**Table 5:** Antibacterial activities of the tested extracts against selected bacterial strains (mg mL^−1^)^a^

Extracts		S.a.	B.c.	L.m.	M.l.	P.ae.	E.c.	S.t.	En.cl.
**Infusion**	**MIC**	2.0	1.0	1.0	3.0	1.5	1.0	2.0	1.0
	**MBC**	4.0	2.0	2.0	4.0	2.0	2.0	4.0	2.0
**70% Ethanolic**	**MIC**	1.0	1.0	1.0	3.0	1.5	0.5	1.0	1.0
	**MBC**	2.0	2.0	2.0	4.0	2.0	1.0	2.0	2.0
**Streptomycin**	**MIC**	0.100	0.025	0.150	0.050	0.100	0.100	0.100	0.025
	**MBC**	0.200	0.050	0.300	0.100	0.200	0.200	0.200	0.050
**Ampicillin**	**MIC**	0.100	0.100	0.150	0.100	0.300	0.150	0.100	0.100
	**MBC**	0.150	0.150	0.500	0.150	0.500	0.200	0.200	0.150

a Minimum inhibitory concentration (MIC) and minimum bactericidal concentration (MBC) values. S.a.: *Staphylococcus aureus*; B.c.: *Bacillus cereus*; M.l.: *Micrococcus luteus*; P.a.: *Pseudomonas aeruginosa*; E.c.: *Escherichia coli*; S.t.: *Salmonella Typhimurium*; En.cl: *Enterobacter cloacae*.

### 3.5 Antifungal activity

Table 6 summarizes the antifungal effects of *Berberis turcomanica* berry 70% ethanolic and infusion extracts against eight pathogenic fungi from *Aspergillus*, *Trichoderma* and *Penicillium* genera. The 70% ethanolic extract generally showed greater antifungal potency, with MIC values ranging from 1.0 to 8.0 mg mL^−1^, compared to 1.5 to 8.0 mg mL^−1^ for the infusion. Particularly notable was the 70% ethanolic extract's activity against *Trichoderma viride* (MIC: 1.0 mg mL^−1^) and *A. versicolor* (MIC: 2.0 mg mL^−1^), both of which showed two-fold lower MICs than the infusion. The 70% ethanolic extract also exhibited consistently lower MFC values across most fungal strains, indicating greater fungicidal activity than the infusion extract. The enhanced antifungal activity of the 70% ethanolic extract may be related to its greater ability to extract moderately polar antifungal phytochemicals, including flavonoids and isoquinoline alkaloids identified in the phytochemical analysis.^15,16^ The plant extracts' detectable antifungal activity against multiple tested fungal strains was observed, especially against dermatomycetes and *Penicillium* species, where the infusion still achieved MICs as low as 1.5 mg mL^−1^. However, comparisons with standard antifungal drugs should be interpreted cautiously because crude plant extracts contain complex mixtures of compounds that may act synergistically rather than as single purified active agents. In comparison with other species, *Berberis vulgaris* root and bark extracts have previously shown antifungal activity with MICs in the range of 0.5–4.0 mg mL^−1^, particularly due to their high berberine content.^54–56^ Additionally, *Berberis aristata* extracts have been shown to exhibit inhibitory effects on *Trichophyton* and *Aspergillus* species, with minimum inhibitory doses ranging from 2.0 to 4.0 mg mL^−1^^56,57^. Previous investigations on *Berberis* species have similarly associated antifungal effects with berberine-rich alkaloid fractions and phenolic constituents, supporting the antifungal relevance of phytochemicals across the genus.^56,58^ The results indicate that the extracts exhibit antifungal effects comparable to those reported for other *Berberis* species, and the higher efficacy of the 70% ethanolic extract further supports improved extraction of less polar bioactive constituents in hydroalcoholic solvents. This observation is consistent with reports indicating that hydroalcoholic solvents improve the recovery of antimicrobial phytochemicals because of their intermediate polarity and broader solubilization capacity for bioactive compounds.^51,53^ These results suggest that BTB may represent a source of bioactive compounds for further phytochemical and biological investigation, although its antifungal activity remains substantially lower than that of conventional antifungal agents.

**Table 6:** Antifungal activity of the tested extracts (mg mL^−1^)^a^

Extracts		A.f.	A.n.	A.v.	A.fl.	T.v.	P.f.	P.o.	P.v.c.
**Infusion**	**MIC**	4.0	8.0	4.0	2.0	1.5	2.0	1.5	1.5
	**MFC**	8.0	≥8.0	8.0	4.0	2.0	4.0	2.0	2.0
**70% Ethanolic**	**MIC**	2.0	8.0	2.0	1.5	1.0	2.0	1.5	1.5
	**MFC**	4.0	≥8.0	4.0	2.0	2.0	4.0	2.0	2.0
**Bifonazole**	**MIC**	0.150	0.150	0.100	0.150	0.150	0.200	0.200	0.100
	**MFC**	0.200	0.200	0.200	0.200	0.200	0.250	0.250	0.200
**Ketoconazole**	**MIC**	0.200	0.200	0.200	0.200	1.000	0.200	1.000	0.200
	**MFC**	0.500	0.500	0.500	0.500	1.500	0.500	1.500	0.300

a Minimum inhibitory concentration (MIC) and minimum fungicidal concentration (MFC) values. A.f.: *Aspregillus fumigatus*; A.n.: *Aspergillus niger*; A.v.: *Aspergillus versicolor*; A.fl.: *Aspergillus flavus*; T.v: *Trichoderma viride*; P.f.: *Penicillium funiculosum*; P.o.: *Penicillium ochrochloron*; P.v.c.: *Penicillium verrucosum* var. *Cyclopium*.

### 3.6 DFT calculations

The parameters introduced to the literature within the framework of conceptual density functional theory are frequently considered not only in the analysis of the chemical reactivities of molecules but also in the prediction of their biological activities.^59^ These parameters, which are related to the electron density of molecules, also provide the possibility of predicting the strength of interaction between the chemical systems under study and biological structures such as enzymes and proteins. According to molecular orbital theory, frontier molecular orbital energies can be used for the prediction of the electron transfer capability of molecules. The molecules with higher HOMO energy level are good electron donors while the molecules with low LUMO energy are good electron acceptors. Those with the highest HOMO energy values are isorhamnetin, kaempferol, quercetin and quercetin 3-*O*-rhamnoside (Table S2 and Fig. S1). Kaempferol 3-*O*-glucoside and quercetin 3-*O*-glucoside and isorhamnetin have the lowest LUMO energy values.

Chemical hardness,^22,60^ a concept introduced by Pearson^61^ in the 1960s and known as the resistance of chemical species to polarization, is a useful CDFT parameter not only for predicting the products formed in chemical reactions but also for comparing the stability of molecules. The relation between hardness and stability is explained through Maximum Hardness Principle.^62^ In short, this principle states that hard chemical systems exhibit high stability. In the light of the calculated chemical hardness values for the major molecular components of the studied extracts, it can be said that Dihydrokaempferol, *p*-Coumaric acid and Quercetin 3-*O*-glucoside molecules are relatively harder and therefore more stable compared to other molecules. On the other hand, among the molecules examined, the most reactive molecule with the lowest hardness is Isorhamnetin. The electrophilicity index reflects the tendency of electrophiles to accept electron pairs from electron-rich nucleophiles. The minimum electrophilicity principle,^63^ an electronic structural principle related to this concept, argues that the electrophilicity index is minimized in steady-state conditions. The principle of minimum electrophilicity, based on the calculated first and second electrophilicity index values, also predicts that the most reactive molecule is Isorhamnetin. In molecular docking analysis section where the interactions with therapeutic enzyme targets – acetylcholinesterase (AChE), butyrylcholinesterase (BChE), amylase, tyrosinase, and glucosidase of twelve molecules, it is seen that quercetin, isorhamnetin and kaempferol have proven more prominent in AChE inhibition. This efficacy can be explained by the low chemical hardness and high reactivity of the molecule in question, as reported in our previous studies.^64^ It is noteworthy that the rutin molecule, which has the most negative docking score in terms of BChE inhibition. In the analyses regarding to amylase inhibition studies it is shown that molecules with high chemical hardness values, such as 3-*O*-caffeoylquinic acid, 5-*O*-caffeoylquinic acid, quercetin 3-*O*-glucoside, and quercetin 3-*O*-rhamnoside, are more effective in inhibition and interact more strongly with the relevant biological system. Calculated docking score implies that 5-*O*-caffeoylquinic acid will be effective than others in Tyrosinase inhibition also. In glucosidase inhibition analysis, 5-*O*-Caffeoylquinic acid, dihydrokaempferol and quercetin molecules interact more strongly with the relevant biological system.

### 3.7 Molecular docking

The binding affinities of 12 selected molecules against five key therapeutic enzyme targets – acetylcholinesterase (AChE), butyrylcholinesterase (BChE), amylase, tyrosinase, and glucosidase – were evaluated *via* molecular docking. The docking scores, expressed as docking scores (kcal mol^−1^), are summarized in the table and reflect the thermodynamic stability of the ligand-protein complexes (Table 7). Overall, the selected molecules exhibited significant inhibitory potential, with several molecules showing multi-target activity. Redocking validation of the crystallographic ligands yielded docking scores of −81.08 for AChE (RMSD: 0.678 Å), −59.45 for BChE (RMSD: 0.332 Å), −74.73 for AML (RMSD: 2.237 Å), and −58.68 for GLC (RMSD: 2.293 Å), confirming the reliability of the docking protocol in reproducing the native binding poses. For TYR, the control ligand Arbutin exhibited a binding score of −80.54 kcal mol^−1^.

**Table 7:** Calculated docking scores (kcal mol^−1^) for selected twelve molecules

Enzymes/molecule name	AChE	BChE	Amylase	Tyrosinase	Glucosidase
3-*O*-Caffeoylquinic acid	−64.04	−76.64	−72.09	−62.35	−79.90
5-*O*-Caffeoylquinic acid	−74.72	−89.95	−74.41	−77.75	−92.64
Caffeic acid	−79.79	−66.03	−65.09	−67.39	−68.61
Dihydrokaempferol	−87.74	−83.26	−67.11	−64.28	−86.42
Isorhamnetin	−83.95	−84.78	−67.44	−67.71	−81.17
Kaempferol	−89.96	−83.35	−64.59	−66.42	−82.54
Kaempferol 3-*O*-glucoside	−65.74	−96.31	−69.51	−65.64	−81.57
*p*-Coumaric acid	−74.62	−65.45	−59.52	−64.75	−64.24
Quercetin	−94.72	−83.19	−68.93	−70.06	−87.45
Quercetin 3-*O*-glucoside	−62.46	−98.96	−75.09	−71.99	−84.05
Quercetin 3-*O*-rhamnoside	−44.22	−88.57	−75.71	−68.60	−82.13
Rutin	−32.06	−104.51	−71.88	−59.20	−61.13

The molecular docking results revealed that the tested polyphenols exhibited high affinity for both AChE and BChE. Quercetin emerged as the strongest ligand for AChE with a docking score of −94.72 kcal mol^−1^, closely followed by kaempferol (−89.96 kcal mol^−1^) and dihydrokaempferol (−87.74 kcal mol^−1^). Interestingly, the affinity profile changed significantly for BChE. Quercetin emerged as the strongest ligand for AChE with a docking score of −94.72 kcal mol^−1^, closely followed by kaempferol (−89.96 kcal mol^−1^) and dihydrokaempferol (−87.74 kcal mol^−1^). Interestingly, the affinity profile changed significantly for BChE. Rutin showed the highest binding stability to BChE (−104.51 kcal mol^−1^), followed by quercetin 3-*O*-glucoside (−98.96 kcal mol^−1^) and kaempferol 3-*O*-glucoside (−96.31 kcal mol^−1^). The increased affinity of glycosylated flavonoids for BChE suggests that the sugar moieties may undergo additional hydrogen bonds or van der Waals interactions in the larger acyl pocket of BChE compared to AChE. Based on the docking analysis against the glucosidase enzyme, 5-*O*-caffeoylquinic acid and quercetin emerged as the top-performing molecules, exhibiting binding energies of −92.64 kcal mol^−1^ and −87.45 kcal mol^−1^, respectively. The high affinity of chlorogenic acid derivatives (caffeoylquinic acids) indicates a strong potential for inhibiting glucose absorption. For amylase, the docking scores were relatively homogeneous across the tested molecules; quercetin 3-*O*-rhamnoside (−75.71 kcal mol^−1^) and quercetin 3-*O*-glucoside (−75.09 kcal mol^−1^) displayed the most favorable interaction energies. Binding scores for tyrosinase ranged from −59.20 to −77.75 kcal mol^−1^. Among the evaluated molecules, 5-*O*-caffeoylquinic acid exhibited the strongest inhibitory potential with a binding energy of −77.75 kcal mol^−1^, followed by quercetin (−70.06 kcal mol^−1^) and quercetin-3-*O*-glucoside (−71.99 kcal mol^−1^).

**Fig. 2:**
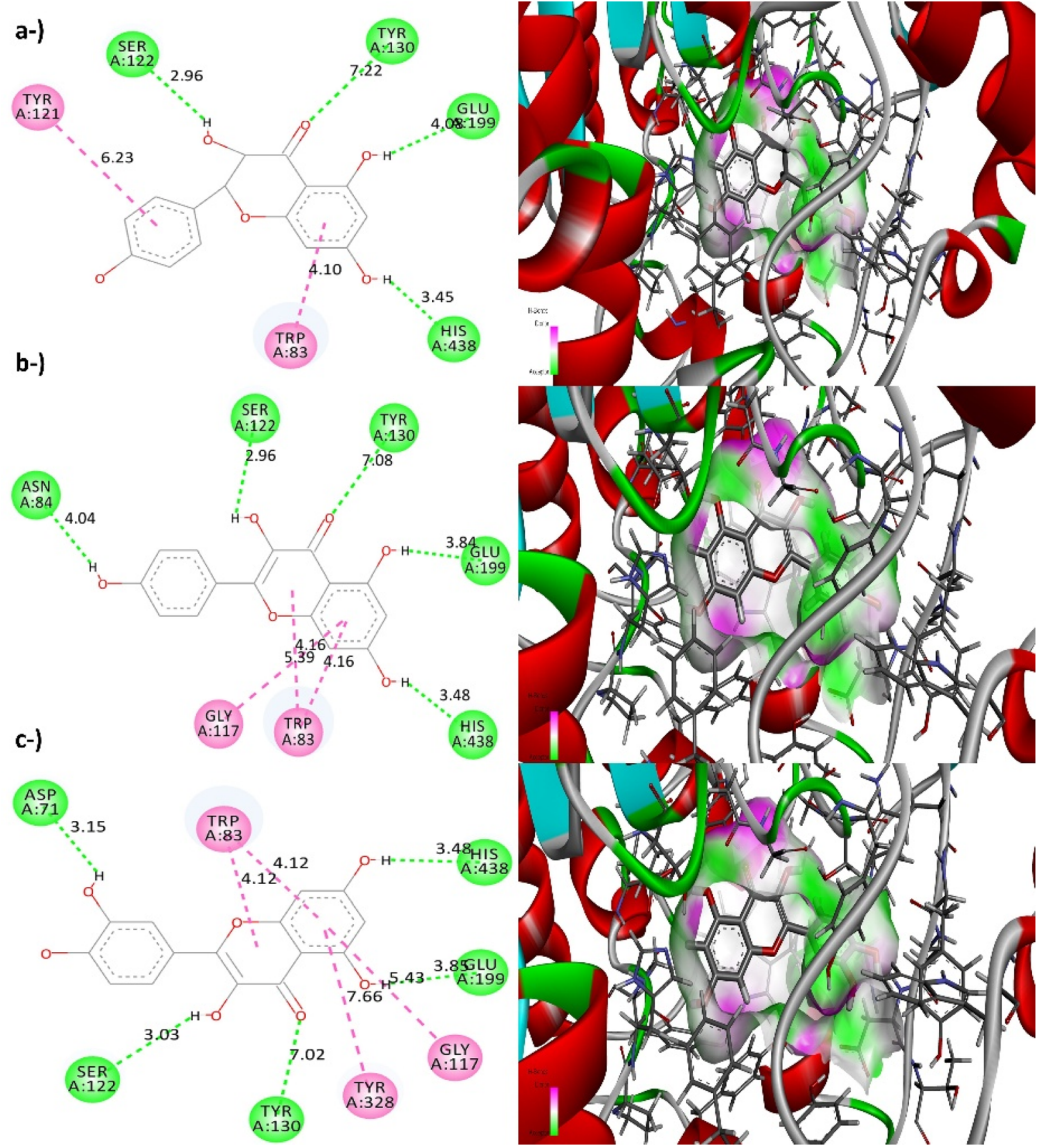
Detailed two and three-dimensional representation of three selected molecules bound within the acetylcholinesterase (AChE) binding site (PDB ID: 1QTI). (a-) Dihydrokaempferol, (b-) kaempferol, (c-) quercetin. (green: Hydrogen bond, purple: pi–pi interaction).

Molecular docking analysis at the acetylcholinesterase (AChE) binding site revealed that the three molecules with the best docking scores - dihydrokaempferol (a), kaempferol (b), and quercetin (c) – occupy the binding site through a complex network of noncovalent interactions (Fig. 2). Although all three ligands are fixed by a conserved hydrogen bond framework containing critical residues such as Ser122, Tyr130, Glu199, and His438, quercetin exhibits the strongest inhibitory profile thanks to an additional hydrogen bond formed between its hydroxyl group and Asp71. Additionally, these three molecules have formed a pi–pi interaction with Trp83.

**Fig. 3:**
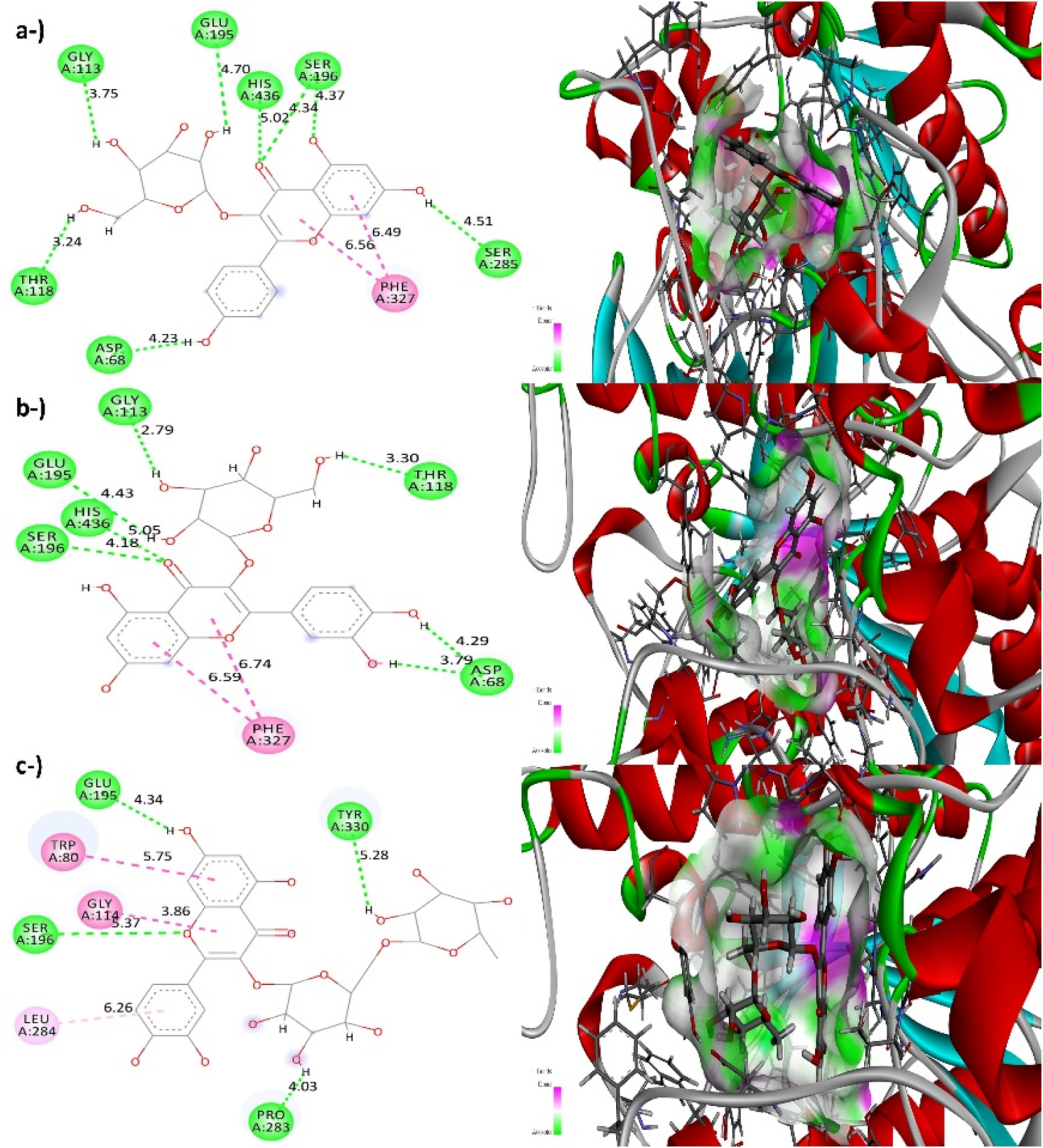
Detailed two and three-dimensional representation of three selected molecules bound within the butyrylcholinesterase (BChE) binding site (PDB ID: 1XLW). (a-) Kaempferol 3-*O*-glucoside, (b-) quercetin 3-*O*-glucoside, (c-) rutin. (green: Hydrogen bond, pink: pi–alkyl interaction, purple:pi–pi interaction).

The molecular docking analysis within the butyrylcholinesterase (BChE) binding site demonstrates that kaempferol 3-*O*-glucoside, quercetin 3-*O*-glucoside, and rutin are effectively stabilized through an extensive network of hydrogen bonds and hydrophobic interactions, primarily facilitated by their glycosidic moieties (Fig. 3). As illustrated in the interaction diagrams, all three glycosylated flavonoids establish a strong hydrogen-bonding framework with key active site residues, including Glu195, and Ser196 which are crucial for the stabilization of the ligand within the binding site. Rutin is stabilized particularly by additional hydrogen bonds with Tyr330 and Pro283, and by pi-alkyl and pi–pi interactions with Trp80 and Gly114. Similarly, kaempferol 3-*O*-glucoside and quercetin 3-*O*-glucoside are further stabilized by pi–pi stacked interactions with Phe327, while their glycone parts extend toward the peripheral site to engage residues like Thr118 and Asp68.

**Fig. 4:**
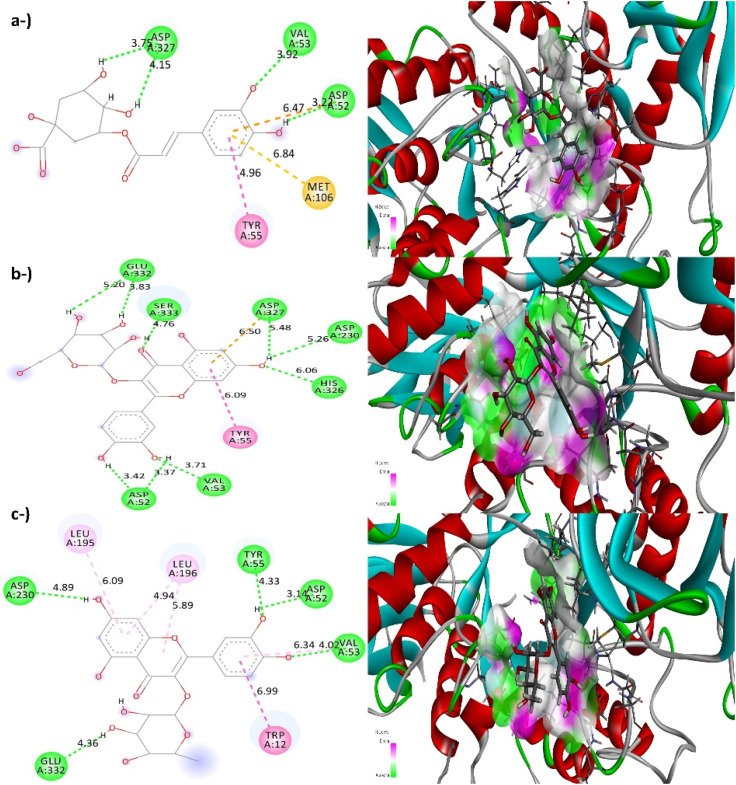
Detailed two and three-dimensional representation of three selected molecules bound within the amylase binding site (PDB ID: 9FZ3). (a-) 5-*O*-Caffeoylquinic acid, (b-) quercetin 3-*O*-glucoside, (c-) quercetin 3-*O*-rhamnoside. (green: Hydrogen bond, pink: pi–alkyl interaction, purple: pi–pi interaction, orange: pi–sulfur interaction).

The molecular docking simulations for amylase reveal that 5-*O*-caffeoylquinic acid, quercetin 3-*O*-glucoside, and quercetin 3-*O*-rhamnoside effectively occupy the binding site, forming stable complexes stabilized by an intricate network of hydrophilic and hydrophobic interactions (Fig. 4). As shown in the 2D and 3D interaction graph, 5-*O*-caffeoylquinic acid is fixed with Asp327, Val53, and Asp52 *via* hydrogen bonds; this is complemented by pi–pi stacking interaction with Tyr55 and pi-sulfur contact with Met106. Similarly, the glycosylated flavonoids quercetin 3-*O*-glucoside and quercetin 3-*O*-rhamnoside leverage their sugar moieties to establish extensive hydrogen bonding networks with residues such as Glu332, Asp230, and Asp52, effectively “locking” the molecules within the binding site. The phenolic nuclei of these flavonoids are further stabilized through pi interactions with Tyr55, Trp12, and Leu196.

**Fig. 5:**
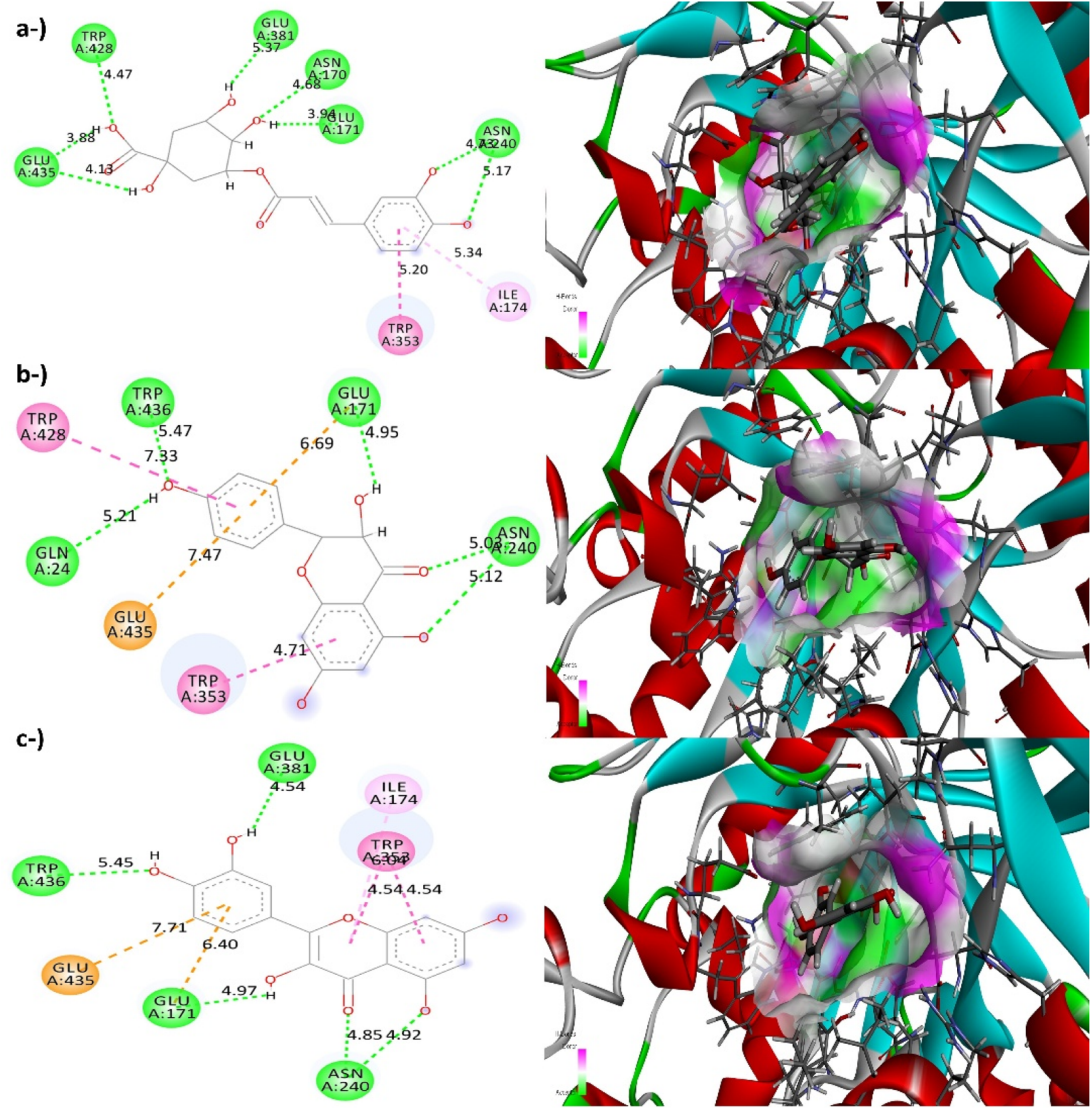
Detailed two and three-dimensional representation of three selected molecules bound within the glucosidase binding site (PDB ID: 2RGL). (a-) 5-*O*-Caffeoylquinic acid, (b-) dihydrokaempferol, (c-) quercetin. (green: Hydrogen bond, pink: pi–alkyl interaction, purple: pi–pi interaction, orange: pi–anion interaction).

Molecular docking analysis against glucosidase reveals that the three molecules with the best docking scores – 5-*O*-caffeoylquinic acid, dihydrokaempferol, and quercetin – are strongly retained in the enzyme's binding site *via* an extensive network of polar and non-polar interactions (Fig. 5). 5-*O*-Caffeoylquinic acid exhibits the most intricate binding orientation, anchored by a dense cluster of hydrogen bonds with residues Glu381, Asn170, Glu171, Asn240, Trp428, and Glu435, while its aromatic moiety is stabilized by pi–pi stacked interactions with Trp353 and hydrophobic contacts with Ile174. In comparison, the flavonoid derivatives dihydrokaempferol and quercetin utilize their polyphenolic scaffolds to engage in critical hydrogen bonding with Glu171, Asn240, and Gln24, complemented by significant pi–anion interactions with Glu435 and pi-stacking with Trp353.

**Fig. 6:**
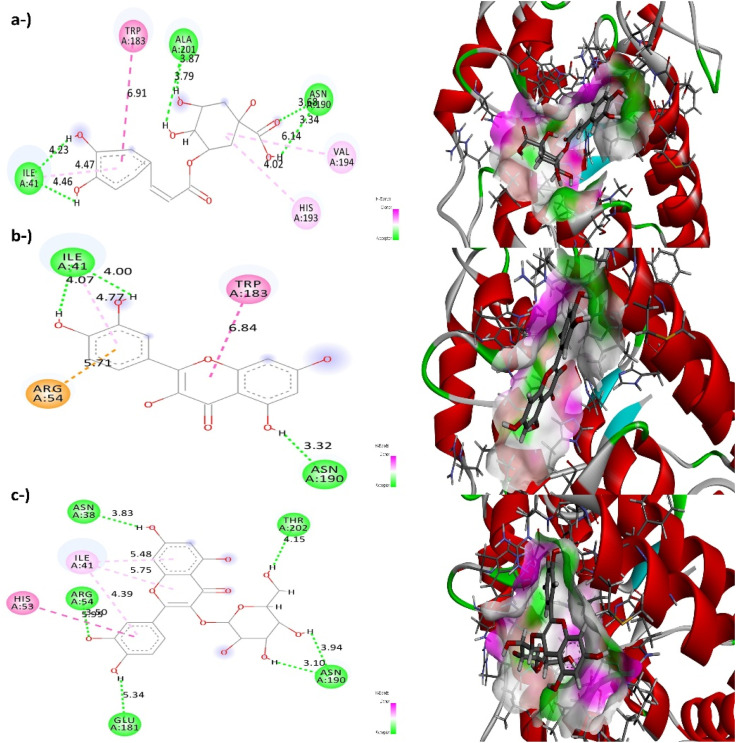
Detailed two and three-dimensional representation of three selected molecules bound within the tyrosinase binding site (PDB ID: 1WX2). (a-) 5-*O*-Caffeoylquinic acid, (b-) quercetin, (c-) quercetin 3-*O*-glucoside. (green: Hydrogen bond, pink: pi–alkyl interaction, purple: pi–pi interaction, orange: pi–cation interaction).

Molecular docking analysis against tyrosinase revealed that 5-*O*-caffeoylquinic acid, quercetin, and quercetin-3-*O*-glucoside are effectively accommodated within the enzyme active site through an intricate network of hydrogen bonds and hydrophobic interactions (Fig. 6). 5-*O*-Caffeoylquinic acid exhibits a highly stabilized pose anchored by a series of hydrogen bonds with residues Ala201, Asn190, and Ile41, while its aromatic rings are further stabilized by pi-stacking with Trp183 and pi-alkyl interactions with His193 and Val194. In contrast, quercetin forms hydrogen bonds with Asn190 and Ile41 using its polyphenolic backbone; these bonds are completed by pi-stacking with Trp183 and pi–cation interaction with Arg54. The glycosylated derivative, Quercetin 3-*O*-glucoside, leverages its glucose moiety to establish additional hydrogen bonds with Thr202, Asn38, and Asn190, while the flavonoid core maintains key contacts with Ile41 and Arg54.

### 3.8 Molecular dynamics simulations and free energy calculations

**Fig. 7:**
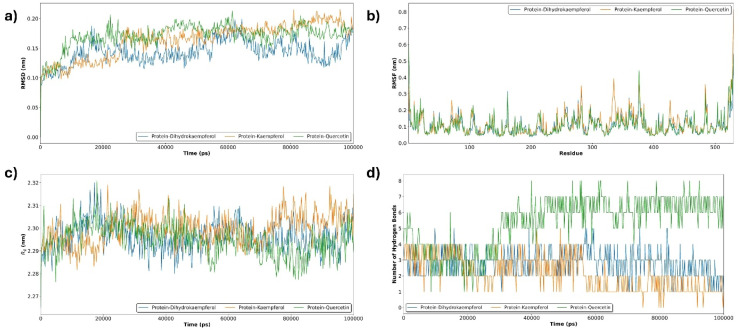
Molecular dynamics simulation analysis of AChE-ligand complexes involving three leading molecules. (a-) root mean square deviation (RMSD), (b-) root mean square fluctuation (RMSF), (c-) radius of gyration, and (d-) hydrogen bond analysis.

Molecular dynamics simulations were conducted on the top three ligand selected for each enzyme based on their highest docking scores, in order to further evaluate the stability and dynamic behavior of the enzyme-ligand complexes. The dynamic stability and thermodynamic binding profiles of dihydrokaempferol, kaempferol, and quercetin in complex with AChE were further obtained through 100 ns molecular dynamics (MD) simulations and free energy calculations. The RMSD trajectories indicate that all complexes reached structural equilibrium after approximately 20 ns, with quercetin exhibiting a slightly higher but stable fluctuation profile around 0.18 nm, suggesting an adaptation within the binding site (Fig. 7a). The RMSF plots show consistent local flexibility across the three complexes, with major fluctuations confined to loop regions, while the binding site residues remained highly stable (Fig. 7b). The Radius of Gyration (*R*_g_) values remained relatively constant throughout the simulation (2.29–2.32 nm), confirming the maintenance of protein compactness upon ligand binding (Fig. 7c). Notably, the hydrogen bond analysis highlights quercetin's superior binding persistence, maintaining a significantly higher number of contacts (up to 7–8 bonds) compared to dihydrokaempferol and kaempferol, which fluctuated between 1 and 4 bonds (Fig. 7d).

This observation is quantitatively supported by the free energy data, where quercetin yielded the most favorable total binding free energy (ΔTOTAL = −40.85 kcal mol^−1^), driven largely by a dominant electrostatic contribution (ΔEEL = −66.16 kcal mol^−1^) (Table 8). In contrast, dihydrokaempferol and kaempferol exhibited lower binding affinities (ΔTOTAL of −28.33 and −26.74 kcal mol^−1^, respectively), primarily due to weaker electrostatic interactions.

**Table 8:** Free energy components of three selected molecules in the complex with acetylcholinesterase (AChE)

Energy component/molecule name	Dihydrokaempferol	Kaempferol	Quercetin
ΔVDWAALS (kcal mol^−1^)	−29.30 ± 0.50	−31.56 ± 0.56	−28.14 ± 0.96
ΔEEL (kcal mol^−1^)	−41.45 ± 3.27	−30.72 ± 7.48	−66.16 ± 4.47
ΔEGB (kcal mol^−1^)	47.07 ± 1.52	39.99 ± 2.96	58.41 ± 1.98
ΔGGAS (kcal mol^−1^)	−70.75 ± 3.30	−62.27 ± 7.50	−94.30 ± 4.57
ΔGSOLV (kcal mol^−1^)	42.42 ± 1.52	35.54 ± 2.96	53.45 ± 1.98
ΔTOTAL (kcal mol^−1^)	−28.33 ± 3.64	−26.74 ± 8.06	−40.85 ± 4.98

**Fig. 8:**
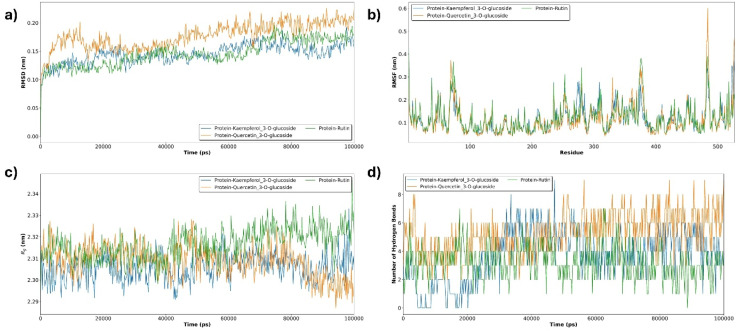
Molecular dynamics simulation analysis of BChE-ligand complexes involving three leading molecules. (a-) root mean square deviation (RMSD), (b-) root mean square fluctuation (RMSF), (c-) radius of gyration, and (d-) hydrogen bond analysis.

The structural stability and thermodynamic binding affinity of kaempferol 3-*O*-glucoside, quercetin 3-*O*-glucoside, and rutin in complex with BChE were rigorously evaluated through 100 ns molecular dynamics (MD) simulations and free energy calculations. The RMSD trajectories reveal that all systems achieved structural equilibrium within the first 30 ns, with quercetin 3-*O*-glucoside and rutin exhibiting stable profiles around 0.15–0.18 nm, indicating high conformational stability within the enzymatic pocket (Fig. 8a). RMSF analysis showed consistent fluctuations along the protein backbone, and no significant fluctuation patterns were observed between complexes (Fig. 8b). The radius of gyration (*R*_g_) remained constant throughout the entire trajectory (approximately 2.30–2.33 nm), confirming the compactness of the enzyme-ligand complexes (Fig. 8c). Hydrogen Bond analysis underscores the superior interaction persistence of quercetin 3-*O*-glucoside, which consistently maintained between 4 and 8 bonds, outperforming the other derivatives (Fig. 8d).

This structural robustness is quantitatively validated by the free energy results, where quercetin 3-*O*-glucoside demonstrated the most favorable binding free energy (ΔTOTAL = −44.52 kcal mol^−1^), significantly driven by superior electrostatic (ΔEEL = −53.02 kcal mol^−1^) and gas-phase (ΔGGAS = −94.71 kcal mol^−1^) contributions (Table 9).

**Table 9:** Free energy components of three selected molecules in the complex with butyrylcholinesterase (BChE)

Energy component/molecule name	Kaempferol 3-*O*-glucoside	Quercetin 3-*O*-glucoside	Rutin
ΔVDWAALS (kcal mol^−1^)	−43.04 ± 1.38	−41.70 ± 1.04	−53.32 ± 1.12
ΔEEL (kcal mol^−1^)	−43.99 ± 3.74	−53.02 ± 4.78	−35.69 ± 6.12
ΔEGB (kcal mol^−1^)	56.44 ± 1.16	56.57 ± 1.58	61.09 ± 2.70
ΔGGAS (kcal mol^−1^)	−87.03 ± 3.99	−94.71 ± 4.89	−89.01 ± 6.22
ΔGSOLV (kcal mol^−1^)	50.17 ± 1.16	50.19 ± 1.58	53.36 ± 2.70
ΔTOTAL (kcal mol^−1^)	−36.86 ± 4.15	−44.52 ± 5.14	−35.65 ± 6.78

**Fig. 9:**
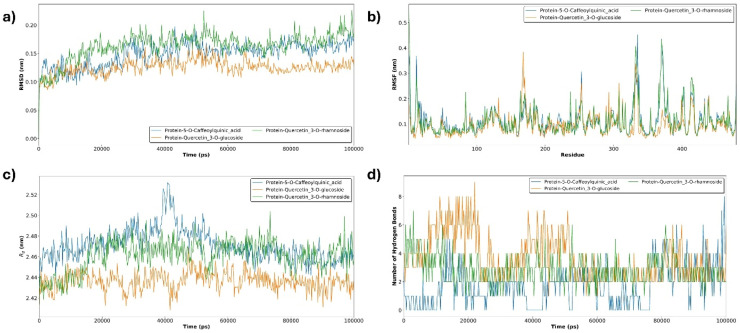
Molecular dynamics simulation analysis of amylase-ligand complexes involving three leading molecules. (a-) root mean square deviation (RMSD), (b-) root mean square fluctuation (RMSF), (c-) radius of gyration, and (d-) hydrogen bond analysis.

The structural stability and binding thermodynamics of 5-*O*-caffeoylquinic acid, quercetin 3-*O*-glucoside, and quercetin 3-*O*-rhamnoside in complex with amylase were comprehensively evaluated through 100 ns molecular dynamics (MD) simulations and free energy calculations. The RMSD values demonstrate that the quercetin 3-*O*-glucoside achieved rapid equilibration with lower average fluctuations (approx. 0.10–0.12 nm) compared to 5-*O*-caffeoylquinic acid, which exhibited a slightly more flexible profile but remained stable after 40 ns (Fig. 9a). RMSF analysis confirms low local flexibility for all complexes, with major fluctuations confined to the solvent-exposed loops while the binding site remained rigid (Fig. 9b). The radius of gyration (*R*_g_) results indicate that quercetin 3-*O*-glucoside maintains the most compact protein-ligand conformation (∼2.44 nm), whereas 5-*O*-caffeoylquinic acid shows a transient expansion around 40 ns before restabilizing (Fig. 9c). Hydrogen Bond analysis highlights the superior interaction persistence of the two quercetin derivatives, which consistently formed between 3 and 8 bonds, whereas the caffeoylquinic acid exhibited more intermittent contact patterns (Fig. 9d).

These dynamic observations are quantitatively supported by free energy data, where quercetin 3-*O*-glucoside and quercetin 3-*O*-rhamnoside yielded significantly higher total binding free energies (ΔTOTAL of −27.04 and −26.20 kcal mol^−1^, respectively) compared to 5-*O*-caffeoylquinic acid (−10.55 kcal mol^−1^) (Table 10).

**Table 10:** Free energy components of three selected molecules in the complex with amylase

Energy component/molecule name	5-*O*-Caffeoylquinic acid	Quercetin 3-*O*-glucoside	Quercetin 3-*O*-rhamnoside
ΔVDWAALS (kcal mol^−1^)	−11.31 ± 0.18	−17.43 ± 1.66	−19.83 ± 1.59
ΔEEL (kcal mol^−1^)	−21.89 ± 0.50	−46.19 ± 4.13	−38.47 ± 1.07
ΔEGB (kcal mol^−1^)	24.84 ± 2.72	40.46 ± 0.92	35.87 ± 0.22
ΔGGAS (kcal mol^−1^)	−33.20 ± 0.53	−63.62 ± 4.45	−58.30 ± 1.91
ΔGSOLV (kcal mol^−1^)	22.65 ± 2.74	36.58 ± 0.92	32.10 ± 0.27
ΔTOTAL (kcal mol^−1^)	−10.55 ± 2.79	−27.04 ± 4.54	−26.20 ± 1.93

**Fig. 10:**
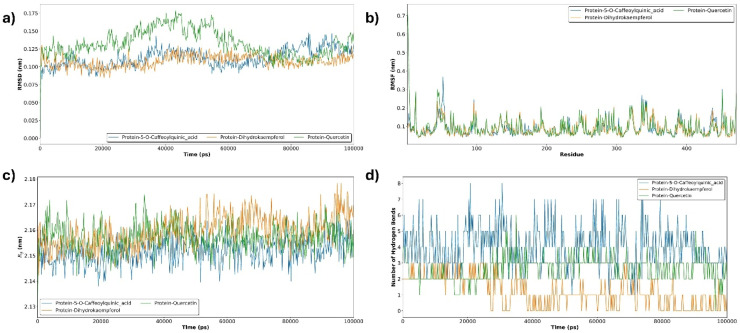
Molecular dynamics simulation analysis of glucosidase-ligand complexes involving three leading molecules. (a-) root mean square deviation (RMSD), (b-) root mean square fluctuation (RMSF), (c-) radius of gyration, and (d-) hydrogen bond analysis.

The structural stability and thermodynamic binding affinity of 5-*O*-caffeoylquinic acid, dihydrokaempferol, and quercetin in complex with glucosidase were comprehensively analyzed through 100 ns molecular dynamics (MD) simulations and free energy calculations. The RMSD values indicate that all complexes achieved a stable equilibrium after 40 ns, with quercetin exhibiting a slightly higher but stable fluctuation profile (∼0.15 nm) compared to 5-*O*-caffeoylquinic acid and dihydrokaempferol (Fig. 10a). RMSF analysis confirms low local flexibility along the enzyme backbone, particularly at the binding site, and no significant fluctuations between complexes were observed (Fig. 10b). The radius of gyration (*R*_g_) remained consistent throughout the entire orbit (2.14–2.17 nm), indicating that a compact spherical structure was maintained upon ligand binding (Fig. 10c). Hydrogen bond analysis highlights the superior interaction network of 5-*O*-caffeoylquinic acid; this acid consistently remains between 3 and 8 bonds, while dihydrokaempferol and quercetin showed a more moderate level of contact (1–4 bonds) Fig. 10d).

These dynamic observations are quantitatively corroborated by the free energy data, where 5-*O*-caffeoylquinic acid yielded the most favorable total binding free energy (ΔTOTAL = −30.21 kcal mol^−1^), significantly driven by a dominant electrostatic contribution (ΔEEL = −50.45 kcal mol^−1^) and van der Waals interactions (ΔVDWAALS = −29.27 kcal mol^−1^) (Table 11). Although quercetin also demonstrated strong affinity (Delta TOTAL = −27.98 kcal mol^−1^), the superior anchoring of the caffeoylquinic acid derivative - facilitated by its extensive hydrogen-bonding network - establishes it as the most thermodynamically stable inhibitor within the glucosidase binding site.

**Table 11:** Free energy components of three selected molecules in the complex with glucosidase

Energy component/molecule name	5-*O*-Caffeoylquinic acid	Dihydrokaempferol	Quercetin
ΔVDWAALS (kcal mol^−1^)	−29.27 ± 1.36	−21.51 ± 0.55	−17.97 ± 1.06
ΔEEL (kcal mol^−1^)	−50.45 ± 4.83	−19.14 ± 2.69	−49.87 ± 3.87
ΔEGB (kcal mol^−1^)	54.72 ± 4.52	27.45 ± 2.11	43.24 ± 1.03
ΔGGAS (kcal mol^−1^)	−79.72 ± 5.02	−40.65 ± 2.74	−67.84 ± 4.02
ΔGSOLV (kcal mol^−1^)	49.51 ± 4.52	24.26 ± 2.12	39.86 ± 1.03
ΔTOTAL (kcal mol^−1^)	−30.21 ± 6.75	−16.39 ± 3.47	−27.98 ± 4.15

**Fig. 11:**
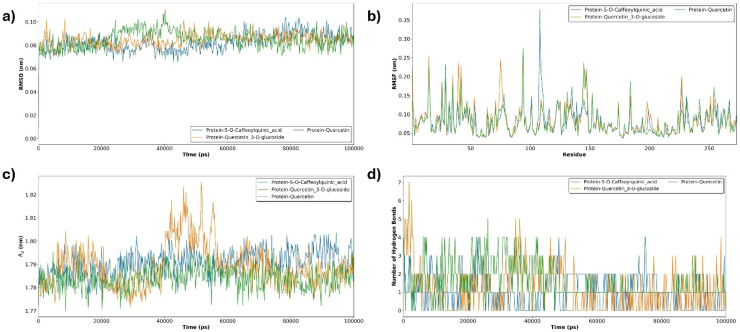
Molecular dynamics simulation analysis of tyrosinase-ligand complexes involving three leading molecules. (a-) root mean square deviation (RMSD), (b-) root mean square fluctuation (RMSF), (c-) radius of gyration, and (d-) hydrogen bond analysis.

The structural integrity and thermodynamic binding preferences of 5-*O*-caffeoylquinic acid, quercetin, and quercetin 3-*O*-glucoside in complex with tyrosinase were rigorously investigated through 100 ns molecular dynamics (MD) simulations and free energy calculations. The RMSD datas reveal that all systems reached a stable plateau after 30 ns, with quercetin 3-*O*-glucoside exhibiting the highest structural stability (lowest fluctuations around 0.08 nm), while quercetin showed a more flexible but equilibrated profile (Fig. 11a). RMSF analysis confirms minimal local fluctuations within the binding site (Fig. 11b), whereas the Radius of Gyration (*R*_g_) remained remarkably consistent throughout the simulation (1.78–1.82 nm), indicating that the enzyme maintained its compact globular architecture upon binding each ligand (Fig. 11c). Hydrogen bond analysis highlighted the interaction continuity of quercetin, which was conserved between 3 and 5 bonds, while 5-*O*-caffeoylquinic acid exhibited a slightly more spaced bonding pattern (Fig. 11d).

These findings are quantitatively supported by the free energy data, where quercetin yielded the most favorable total binding free energy (ΔTOTAL = −19.61 kcal mol^−1^), closely followed by quercetin 3-*O*-glucoside (−11.54 kcal mol^−1^), both significantly outperforming 5-*O*-caffeoylquinic acid (−10.49 kcal mol^−1^) (Table 12). The binding affinity observed for the quercetin derivatives is fundamentally driven by a synergistic combination of potent electrostatic (ΔEEL) and van der Waals (ΔVDW) forces.

**Table 12:** Free energy components of three selected molecules in the complex with tyrosinase

Energy component/molecule name	5-*O*-Caffeoylquinic acid	Quercetin	Quercetin 3-*O*-glucoside
ΔVDWAALS (kcal mol^−1^)	−14.40 ± 0.47	−23.07 ± 0.60	−19.24 ± 1.56
ΔEEL (kcal mol^−1^)	−11.19 ± 4.55	−19.32 ± 3.18	−15.73 ± 5.33
ΔEGB (kcal mol^−1^)	17.37 ± 1.88	26.12 ± 1.10	26.34 ± 1.57
ΔGGAS (kcal mol^−1^)	−25.59 ± 4.57	−42.38 ± 3.23	−34.97 ± 5.55
ΔGSOLV (kcal mol^−1^)	15.11 ± 1.89	22.77 ± 1.10	23.43 ± 1.58
ΔTOTAL (kcal mol^−1^)	−10.49 ± 4.95	−19.61 ± 3.42	−11.54 ± 5.77

## 4 Conclusions

This study provides the first comprehensive pharmacological and phytochemical evaluation of *Berberis turcomanica* berries from Turkmenistan. The infusion and 70% ethanolic extracts of BTB demonstrated strong antioxidant potential, significant enzyme inhibitory activities, and notable antibacterial and antifungal effects, while also exhibiting good biosafety toward human HaCaT cell lines. These bioactivities are supported by the high content of phenolic and flavonoid compounds, along with the presence of other bioactive phytochemicals. The BTB infusion and its 70% ethanolic extract exhibited bioactive constituents with potent antibacterial and antifungal properties, demonstrating efficacy against a broad spectrum of microbial pathogens. The results suggest that BTB are a promising natural source of multifunctional agents with potential applications in the development of pharmaceutical and nutraceutical products. Further studies focusing on the isolation and characterization of major phenolic acids and flavonoid compounds, particularly caffeoylquinic acid derivatives, quercetin glycosides, and berberine-related alkaloids, as well as *in vivo* antioxidant, anti-inflammatory, and antimicrobial models, are warranted to better clarify the therapeutic potential of *B. turcomanica* berry extracts.

## Author contributions

Serdar Korpayev: investigation, methodology, formal analysis, writing – original draft, writing – review and editing. Emre Can Buluz: investigation, methodology, visualization, formal analysis. Jasmina Glamočlija: investigation, methodology, writing – original draft, writing – review and editing. Neda Popović: investigation, methodology, writing – original draft, writing – review and editing. Uroš Gašić: investigation, methodology, writing – original draft, writing – review and editing. Dejan Stojković: investigation, methodology, writing – original draft, writing – review and editing. Hemra Hamrayev: investigation, methodology, resources. Mirap Agamuradov: investigation, methodology, resources. Savaş Kaya: investigation, methodology, visualization, formal analysis. Enver Saka: investigation, methodology, formal analysis, writing – original draft, writing – review and editing. Gökhan Zengin: investigation, methodology, formal analysis, writing – original draft, writing – review and editing.

## Conflicts of interest

There are no conflicts to declare.

## Supplementary Material

RA-016-D6RA02922A-s001

## Data Availability

Data will be requested from authors. Supplementary information (SI) is available. See DOI: https://doi.org/10.1039/d6ra02922a.

## References

[cit1] Paniagua-Zambrana N. Y. , Bussmann R. W., Kikvidze Z. and Khojimatov O. K., in Ethnobotany of the Mountain Regions of Eastern Europe: Carpathians, Springer, 2024, pp. 1–15

[cit2] Rahimi-Madiseh M., Lorigoini Z., Zamani-Gharaghoshi H., Rafieian-Kopaei M. (2017). Iran. J. Basic Med. Sci..

[cit3] Gurbanguly B. , Tükmen Döwlet Neşirýat Gullugy, 2010

[cit4] Bhardwaj D., Kaushik N. (2012). Phytochem. Rev..

[cit5] Khamidov I., Aripova S., Telezhenetskaya M., Faskhutdinov M., Karimov A., Dzhepberov I. (1996). Chem. Nat. Compd..

[cit6] Karimov A., Levkovich M., Abdullaev N., Shakirov R. (1993). Chem. Nat. Compd..

[cit7] El-Zahar K. M., Al-Jamaan M. E., Al-Mutairi F. R., Al-Hudiab A. M., Al-Einzi M. S., Mohamed A. A.-Z. (2022). Molecules.

[cit8] Imanshahidi M., Hosseinzadeh H. (2008). Phytother Res..

[cit9] Kalmarzi R. N., Naleini S. N., Ashtary-Larky D., Peluso I., Jouybari L., Rafi A., Ghorat F., Heidari N., Sharifian F., Mardaneh J. (2019). Oxid. Med. Cell. Longev..

[cit10] Rad S. Z. K., Rameshrad M., Hosseinzadeh H. (2017). Iran. J. Basic Med. Sci..

[cit11] Campisi A., Acquaviva R., Bonfanti R., Raciti G., Amodeo A., Mastrojeni S., Ragusa S., Iauk L. (2014). Sci. World J..

[cit12] Bhatt L. R., Wagle B., Adhikari M., Bhusal S., Giri A., Bhattarai S. (2018). Pharmacogn. J..

[cit13] Nikitin V. V. e. and Gel′dikhanov A. M., Opredelitel′ Rasteniĭ Turkmenistana, Nauka, Leningradskoe otd-nie, 1988

[cit14] Imenshahidi M., Hosseinzadeh H. (2016). Phytother Res..

[cit15] Chaves J. O., de Souza M. C., da Silva L. C., Lachos-Perez D., Torres-Mayanga P. C., Machado A., Forster-Carneiro T., Vázquez-Espinosa M., González-de-Peredo A. V., Barbero G. F., Rostagno M. A. (2020). Front. Chem..

[cit16] Dirar A. I., Alsaadi D. H. M., Wada M., Mohamed M. A., Watanabe T., Devkota H. P. (2019). South Afr. J. Bot..

[cit17] Korpayev S., Zengin G., Glamočlija J., Soković M., Aničić N., Gašić U., Stojković D., Agamuradov M., Agamyradova G. (2025). Chem. Biodiversity.

[cit18] Radović M., Milatović D., Tešić Ž., Tosti T., Gašić U., Dojčinović B., Zagorac D. D. (2020). J. Food Compos. Anal..

[cit19] Korpayev S., Hamrayev H., Aničić N., Gašić U., Zengin G., Agamyradov M., Agamyradova G., Rozyyev H., Amanov G. (2024). Biomass Convers. Biorefinery.

[cit20] Korpayev S., Zengin G., Ak G., Glamočlija J., Soković M., Aničić N., Gašić U., Stojković D., Agamyradov M., Cetiz M. V. (2025). Food Sci. Nutr..

[cit21] Neese F. (2022). Wiley Interdiscip. Rev.: Comput. Mol. Sci..

[cit22] Kaya S., Işın D. Ö., Karakuş N. (2022). J. Indian Chem. Soc..

[cit23] Kaya S., Putz M. V. (2022). Molecules.

[cit24] Parr R. G., Szentpály L. v., Liu S. (1999). J. Am. Chem. Soc..

[cit25] Von Szentpály L. (2000). Int. J. Quantum Chem..

[cit26] Koopmans T. (1934). Physica.

[cit27] Exner T. E., Korb O., Ten Brink T. (2009). Chem. Cent. J..

[cit28] Bartolucci C., Perola E., Pilger C., Fels G., Lamba D. (2001). Proteins: Struct., Funct., Bioinf..

[cit29] Nachon F., Asojo O. A., Borgstahl G. E., Masson P., Lockridge O. (2005). Biochemistry.

[cit30] Matoba Y., Kumagai T., Yamamoto A., Yoshitsu H., Sugiyama M. (2006). J. Biol. Chem..

[cit31] Pickles I. B., Chen Y., Moroz O., Brown H. A., de Boer C., Armstrong Z., McGregor N. G., Artola M., Codée J. D., Koropatkin N. M. (2025). Angew. Chem., Int. Ed..

[cit32] Chuenchor W., Pengthaisong S., Robinson R. C., Yuvaniyama J., Oonanant W., Bevan D. R., Esen A., Chen C.-J., Opassiri R., Svasti J. (2008). J. Mol. Biol..

[cit33] Burley S. K., Bhikadiya C., Bi C., Bittrich S., Chen L., Crichlow G. V., Duarte J. M., Dutta S., Fayazi M., Feng Z. (2022). Protein Sci..

[cit34] DeLano W. L. , https://www.pymol.org/, 2002

[cit35] BIOVA DS, Discovery studio modeling environment, release 2020, Dassault Systemes, San Diego, 2020

[cit36] Lindorff-Larsen K., Piana S., Palmo K., Maragakis P., Klepeis J. L., Dror R. O., Shaw D. E. (2010). Proteins: Struct., Funct., Bioinf..

[cit37] Sousa da Silva A. W., Vranken W. F. (2012). BMC Res. Notes.

[cit38] Valdés-Tresanco M. S., Valdés-Tresanco M. E., Valiente P. A., Moreno E. (2021). J. Chem. Theor. Comput..

[cit39] Alemardan A., Asadi W., Rezaei M., Tabrizi L., Mohammadi S. (2013). Ind. Crops Prod..

[cit40] Bibi N., Shah M. H., Khan N., Al-Hashimi A., Elshikh M. S., Iqbal A., Ahmad S., Abbasi A. M. (2022). Plants.

[cit41] Panche A. N., Diwan A. D., Chandra S. R. (2016). J. Nutr. Sci..

[cit42] Serafini M., Peluso I., Raguzzini A. (2010). Proc. Nutr. Soc..

[cit43] Özgen M., Saraçoğlu O., Geçer E. N. (2012). Hortic. Environ. Biotechnol..

[cit44] Rice-Evans C. A., Miller N. J., Paganga G. (1996). Free Radic. Biol. Med..

[cit45] Ye Z., Wang Q., Dai S., Ji X., Cao P., Xu C., Bao G. (2022). In Vitro Anim. Cell Dev. Biol..

[cit46] Motalleb G. , Hanachi P., Kua S., Fauziah O. and Asmah R., 2005

[cit47] Bathaei P., Imenshahidi M., Hosseinzadeh H. (2025). N. Schmied. Arch. Pharmacol..

[cit48] Chang T.-S. (2009). Int. J. Mol. Sci..

[cit49] Kim Y.-J., Uyama H. (2005). CMLS Cell. Mol. Life Sci..

[cit50] Cushnie T. T., Lamb A. J. (2011). Int. J. Antimicrob. Agents.

[cit51] Daglia M. (2012). Curr. Opin. Biotechnol..

[cit52] Musumeci R., Speciale A., Costanzo R., Annino A., Ragusa S., Rapisarda A., Pappalardo M., Iauk L. (2003). Int. J. Antimicrob. Agents.

[cit53] Abd El-Wahab A. E., Ghareeb D. A., Sarhan E. E., Abu-Serie M. M., El Demellawy M. A. (2013). BMC Compl. Alternative Med..

[cit54] Kazemipoor M., Fadaei Tehrani P., Zandi H., Golvardi Yazdi R. (2021). Clin. Exp. Dent. Res..

[cit55] Zhang C.-W., Huang D.-Y., Rajoka M. S. R., Wu Y., He Z.-D., Ye L., Wang Y., Song X. (2024). Molecules.

[cit56] Singh M., Srivastava S., Rawat A. (2007). Fitoterapia.

[cit57] Shahid M., Rahim T., Shahzad A., Latif T., Fatma T., Rashid M., Raza A., Mustafa S. (2009). Afr. J. Biotechnol..

[cit58] Freile M. L., Giannini F., Pucci G., Sturniolo A., Rodero L., Pucci O., Balzareti V., Enriz R. (2003). Fitoterapia.

[cit59] Poddar A. and Chattaraj P. K., in Materials Informatics I: Methods, Springer, 2025, pp. 139–166

[cit60] Kaya S., Robles-Navarro A., Mejía E., Gómez T., Cardenas C. (2022). J. Phys. Chem..

[cit61] Pearson R. G. (1963). J. Am. Chem. Soc..

[cit62] Parr R. G., Chattaraj P. K. (1991). J. Am. Chem. Soc..

[cit63] von Szentpály L., Kaya S., Karakuş N. (2020). J. Phys. Chem..

[cit64] Gürer E. S., Yıldırım Ş., Kocyigit Ü. M., Berisha A., Kaya S. (2025). J. Mol. Struct..

