# Supplementary material for: Berberis turcomanica berries: an integrated evaluation of antioxidant, enzyme inhibitory, and antimicrobial activities, phytochemical profile and in silico analysis
Source: RSC Adv. 2026 Jun 5;16(33):30814–32. doi: 10.1039/d6ra02922a (PMC13245373; doi:10.1039/d6ra02922a)
Supplement: RA-016-D6RA02922A-s001 [file RA-016-D6RA02922A-s001.pdf]

Supplementary Information (SI) for RSC Advances.
This journal is © The Royal Society of Chemistry 2026

Assays for Total Phenolic and Flavonoid Contents

The total phenolic content was determined by employing the methods given in the literature with some modification. Sample solution (0.25 mL) was mixed with diluted Folin–Ciocalteu reagent (1 mL, 1:9, v/v) and shaken vigorously. After 3 min, Na_2CO_3 solution (0.75 mL, 1%) was added and the sample absorbance was read at 760 nm after a 2 h incubation at room temperature. The total phenolic content was expressed as milligrams of gallic acid equivalents (mg GAE/g extract)[1].

The total flavonoid content was determined using the $AlCl_3$ method. Briefly, sample solution (1 mL) was mixed with the same volume of aluminum trichloride (2%) in methanol. Similarly, a blank was prepared by adding sample solution (1 mL) to methanol (1 mL) without $AlCl_3$. The sample and blank absorbances were read at 415 nm after a 10 min incubation at room temperature. The absorbance of the blank was subtracted from that of the sample. Rutin was used as a reference standard and the total flavonoid content was expressed as milligrams of rutin equivalents (mg RE/g extract) [1].

Determination of Antioxidant and Enzyme Inhibitory Effects

Antioxidant (DPPH and ABTS radical scavenging, reducing power (CUPRAC and FRAP), phosphomolybdenum and metal chelating (ferrozine method)) and enzyme inhibitory activities (cholinesterase (Elmann's method), tyrosinase (dopachrome method), α-amylase (iodine/potassium iodide method), α - glucosidase (chromogenic PNPG method) and pancreatic lipase (*p*-nitrophenyl butyrate (p-NPB) method) were determined using the methods previously described by Uysal et al. [1] and Grochowski et al. [2]

For the DPPH (1,1-diphenyl-2-picrylhydrazyl) radical scavenging assay: Sample solution was added to 4 mL of a 0.004% methanol solution of DPPH. The sample absorbance was read at 517 nm after a 30 min incubation at room temperature in the dark. DPPH radical scavenging activity was expressed as milligrams of trolox equivalents (mg TE/g extract).

For ABTS (2,2′-azino-bis(3-ethylbenzothiazoline) 6-sulfonic acid) radical scavenging assay: Briefly, ABTS+ was produced directly by reacting 7 mM ABTS solution with 2.45 mM potassium persulfate and allowing the mixture to stand for 12–16 in the dark at room temperature. Prior to beginning the assay, ABTS solution was diluted with methanol to an absorbance of 0.700 ± 0.02 at 734 nm. Sample solution was added to ABTS solution (2 mL) and mixed. The sample absorbance was read at 734 nm after a 30 min incubation at room temperature. The ABTS radical scavenging activity was expressed as milligrams of trolox equivalents (mg TE/g extract).

For CUPRAC (cupric ion reducing activity) activity assay: Sample solution was added to premixed reaction mixture containing $CuCl_2$ (1 mL, 10 mM), neocuproine (1 mL, 7.5 mM) and NH_4Ac buffer (1 mL, 1 M, pH 7.0). Similarly, a blank was prepared by adding sample solution (0.5 mL) to premixed reaction mixture (3 mL) without $CuCl_2$. Then, the sample and blank absorbances were read at 450 nm after a 30 min incubation at room temperature. The absorbance of the blank was subtracted from that of the sample. CUPRAC activity was expressed as milligrams of trolox equivalents (mg TE/g extract).

For FRAP (ferric reducing antioxidant power) activity assay: Sample solution was added to premixed FRAP reagent (2 mL) containing acetate buffer (0.3 M, pH 3.6), 2,4,6-tris(2-pyridyl)-S-triazine (TPTZ) (10 mM)

in 40 mM HCl and ferric chloride (20 mM) in a ratio of 10:1:1 (v/v/v). Then, the sample absorbance was read at 593 nm after a 30 min incubation at room temperature. FRAP activity was expressed as milligrams of trolox equivalents (mg TE/g extract).

For phosphomolybdenum method: Sample solution was combined with 3 mL of reagent solution (0.6 M sulfuric acid, 28 mM sodium phosphate and 4 mM ammonium molybdate). The sample absorbance was read at 695 nm after a 90 min incubation at 95 °C. The total antioxidant capacity was expressed as millimoles of trolox equivalents (mmol TE/g extract).

For metal chelating activity assay: Briefly, sample solution was added to $FeCl_2$ solution (0.05 mL, 2 mM). The reaction was initiated by the addition of 5 mM ferrozine (0.2 mL). Similarly, a blank was prepared by adding sample solution (2 mL) to $FeCl_2$ solution (0.05 mL, 2 mM) and water (0.2 mL) without ferrozine. Then, the sample and blank absorbances were read at 562 nm after 10 min incubation at room temperature. The absorbance of the blank was sub-tracted from that of the sample. The metal chelating activity was expressed as milligrams of EDTA (disodium edetate) equivalents (mg EDTAE/g extract).

For Cholinesterase (ChE) inhibitory activity assay: Sample solution (was mixed with DTNB (5,5-dithio-bis(2-nitrobenzoic) acid, Sigma, St. Louis, MO, USA) (125 µL) and AChE (acetylcholines-terase (Electric ell acetylcholinesterase, Type-VI-S, EC 3.1.1.7,Sigma)), or BChE (butyrylcholinesterase (horse serum butyrylcholinesterase, EC 3.1.1.8, Sigma)) solution (25 µL) in Tris–HCl buffer (pH 8.0) in a 96-well microplate and incubated for 15 min at 25 °C. The reaction was then initiated with the addition of acetylthiocholine iodide (ATCI, Sigma) or butyrylthiocholine chloride (BTCl, Sigma) (25 µL). Similarly, a blank was prepared by adding sample solution to all reaction reagents without enzyme (AChE or BChE) solution. The sample and blank absorbances were read at 405 nm after 10 min incubation at 25 °C. The absorbance of the blank was subtracted from that of the sample and the cholinesterase inhibitory activity was expressed as galanthamine equivalents (mgGALAE/g extract).

For Tyrosinase inhibitory activity assay: Sample solution was mixed with tyrosinase solution (40 µL, Sigma) and phosphate buffer (100 µL, pH 6.8) in a 96-well microplate and incubated for 15 min at 25 °C. The reaction was then initiated with the addition of L-DOPA (40 µL, Sigma). Similarly, a blank was prepared by adding sample solution to all reaction reagents without enzyme (tyrosinase) solution. The sample and blank absorbances were read at 492 nm after a 10 min incubation at 25 °C. The absorbance of the blank was subtracted from that of the sample and the tyrosinase inhibitory activity was expressed as kojic acid equivalents (mgKAE/g extract).

For α-amylase inhibitory activity assay: Sample solution was mixed with α-amylase solution (ex-porcine pancreas, EC 3.2.1.1, Sigma) (50 µL) in phosphate buffer (pH 6.9 with 6 mM sodium chloride) in a 96-well microplate and incubated for 10 min at 37 °C. After pre-incubation, the reaction was initiated with the addition of starch solution (50 µL, 0.05%). Similarly, a blank was prepared by adding sample solution to all reaction reagents without enzyme (α-amylase) solution. The reaction mixture was incubated 10 min at 37 °C. The reaction was then stopped with the addition of HCl (25 µL, 1 M). This was followed by addition of the iodine-potassium iodide solution (100 µL). The sample and blank absorbances were read at 630 nm. The absorbance of the blank was

subtracted from that of the sample and the α-amylase inhibitory activity was expressed as acarbose equivalents (mmol ACE/g extract).

For α-glucosidase inhibitory activity assay: Sample solution was mixed with glutathione (50 µL), α-glucosidase solution (from Saccharomyces cerevisiae, EC 3.2.1.20, Sigma) (50 µL) in phosphate buffer (pH 6.8) and PNPG (4-N-trophenyl-α-D-glucopyranoside, Sigma) (50 µL) in a 96-well microplate and incubated for 15 min at 37 °C. Similarly, a blank was prepared by adding sample solution to all reaction reagents without enzyme (α-glucosidase) solution. The reaction was then stopped with the addition of sodium carbonate (50 µL, 0.2 M). The sample and blank absorbances were read at 400 nm. The absorbance of the blank was subtracted from that of the sample and the α-glucosidase inhibitory activity was expressed as acarbose equivalents (mmol ACE/g extract).

Antibacterial and antifungal activities

Antibacterial and antifungal activities of *Berberis turcmanica* berry (BTB) extracts were assessed using the modified broth microdilution method. The antibacterial activity was evaluated against a panel of clinically relevant Gram-positive and Gram-negative bacterial strains, including *Staphylococcus aureus*, *Bacillus cereus*, *Micrococcus luteus*, *Listeria monocytogenes*, *Pseudomonas aeruginosa*, *Salmonella enterica* serovar Typhimurium, *Escherichia coli*, and *Enterobacter cloacae*. Antifungal activity was tested against filamentous fungi, namely *Aspergillus fumigatus*, *A. versicolor*, *A. ochraceus*, *A. niger*, *Trichoderma viride*, *Penicillium funiculosum*, *P. ochrochloron*, and *P. verrucosum* var. *cyclopium*. Briefly, serial two-fold dilutions of the extracts (10mg/mL) were prepared in 96-well microtiter plates using appropriate growth media. Following inoculation with standardized microbial suspensions, bacterial plates were incubated at 37 °C for 24 h, while fungal plates were incubated under appropriate conditions (25 °C for 72h). Antimicrobial activity was expressed as the minimum inhibitory concentration (MIC), defined as the lowest extract concentration preventing visible microbial growth, and the minimum bactericidal/fungicidal concentration (MBC/MFC), defined as the lowest concentration resulting in the absence of viable microorganisms after subcultivation. All assays were performed in triplicate. The extraction solvent served as a negative control, while standard antimicrobial agents (streptomycin, ampicillin, bifonazole and ketoconazole) were used as positive controls

Cytotoxicity

The cytotoxicity of the extracts was tested on spontaneously transformed, aneuploidy, immortalized keratinocyte cell line from adult human skin (HaCaT). HaCaT cells (Catalog No. T0020001) were purchased from AddexBio and used for the *in vitro* experiments. This cell line is used as an effective *in vitro* alternative for an initial indicative screening of safety aspects of substances. For this purpose, the crystal violet assay was used with some modifications. The extracts were dissolved in phosphate buffered saline (PBS) to a final concentration of 8 mg/mL. HaCaT cells were grown in high-glucose Dulbecco's Modified Eagle Medium (DMEM) supplemented with fetal bovine serum (FBS, 10%), L-glutamine (2 mM), and antibiotic-antimycotic (1%) at 37 ºC and 5% CO2. Forty-eight hours before treatment, 10^4 cells were seeded per well of a 96-well sticky-bottom microtiter plate. After 48 hours, the medium was removed and fresh medium containg different cells concentrations of the extracts was added to the cells. The cells were treated with the extracts for 24 hours. The medium then was removed, the cells were washed twice with PBS and stained with a 0.4% crystal violet solution for 20 minutes at room temperature. Then, the cells were washed and air-dried at room temperature. The absorbance of the dye dissolved

in methanol was measured at 570 nm (OD570) in a plate reader. The results were expressed as IC_{50} value indicating 50% growth inhibition of HaCaT cells when compared to the untreated control. The assay was performed in triplicate. The solvent was used as a negative control.

Table S1. List of phenolic compounds determined in the investigated extracts; retention times, precursor and product ions with specified collision energies, calibration range, equation parameters, determination coefficient (R^2), LOD (limit of detection), and LOQ (limit of quantification).

**Compounds**	**Retention time, min**	**Precursor ions, *m/z***	**Product ions (*m/z*) with specified collision energies (eV)**	**Calibration range, mg/L**	**Equation,Y = A+B*X**	R^2	**LOD, mg/L**	**LOQ, mg/L**
**3-O-Caffeoylquinic acid**	5.00	353.103	191.28 (25)	0.01-1.00	Y = -1729+513670*X	0.9968	0.07	0.24
**5-O-Caffeoylquinic acid**	5.67	353.101	191.18 (25)	0.01-1.00	Y = -178+770960*X	0.9959	0.08	0.27
**Caffeic acid**	6.18	179.004	135.06 (18); 107.13 (18)	0.05-1.00	Y = 2169830*X	0.9950	0.08	0.28
**Rutin**	6.71	609.197	301.20 (32); 299.98 (42)	0.01-1.00	Y = -8085+4756710*X	0.9994	0.03	0.10
**Vitexin**	6.80	430.999	341.10 (25); 311.06 (27)	0.01-1.00	Y = -16625+8356270*X	0.9978	0.07	0.24
**p-Coumaric acid**	6.96	163.072	119.34 (20)	0.25-1.00	Y = 63215+94534*X	0.9976	0.06	0.19
**Quercetin 3-O-glucoside**	6.97	463.002	301.04 (23); 300.02 (29)	0.01-0.75	Y = -20398+4970500*X	0.9996	0.02	0.07
**Isorhamnetin 3-O-rutinoside**	7.13	622.827	313.95 (7); 299.96 (51)	0.01-1.00	Y = -1172+1290340*X	0.9996	0.02	0.08
**Isorhamnetin 3-O-glucoside**	7.39	477.127	314.05 (15); 300.901 (42)	0.01-0.75	Y = -3980+824761*X	0.9991	0.03	0.10
**Quercetin 3-O-rhamnoside**	7.40	447.316	300.96 (20); 299.65 (16)	0.01-1.00	Y = -6048+2357020*X	0.9996	0.03	0.09
**Kaempferol 3-O-glucoside**	7.41	447.008	284.03 (29); 255.03 (43)	0.01-1.00	Y = 9791+3433970*X	0.9978	0.06	0.20

**Myricetin**	7.90	316.925	179.19 (29); 150.90 (21)	0.10-0.75	Y = -46877+661885*X	0.9921	0.09	0.31
**Dihydrokaempferol**	8.09	287.124	269.06 (20); 259.05 (30)	0.01-1.00	Y = -2091+331512*X	0.9994	0.03	0.11
**Eriodictyol**	8.62	286.974	150.93 (19); 135.02 (22)	0.01-0.75	Y = -25400+2419190*X	0.9963	0.06	0.20
**Luteolin**	8.70	285.035	175.04 (27); 133.06 (36)	0.01-1.00	Y = -10176+3010780*X	0.9953	0.09	0.29
**Quercetin**	8.79	301.055	179.22 (19); 151.03 (20)	0.05-0.75	Y = -34294+2402090*X	0.9975	0.06	0.18
**Naringenin**	9.39	271.036	151.01 (20); 107.07 (26)	0.01-1.00	Y = -29883+2692320*X	0.9993	0.03	0.11
**Apigenin**	9.40	269.032	225.09 (23); 151.00 (26)	0.05-1.00	Y = -4231+705653*X	0.9964	0.08	0.25
**Kaempferol**	9.57	285.074	227.07 (33); 151.05 (25)	0.05-1.00	Y = -1222+94624*X	0.9921	0.11	0.38
**Hispidulin**	9.57	299.056	284.11 (35)	0.01-1.00	Y = 21735+14207700*X	0.9979	0.06	0.19
**Isorhamnetin**	9.73	314.989	299.95 (25)	0.01-0.75	Y = -43722+1639670*X	0.9966	0.06	0.19

Table S2. Calculated structural parameters of the major molecular components of the studied extracts

**Molecule**	**E_{HOMO} (eV)**	**E_{LUMO} (eV)**	**χ**	**η**	**σ**	**ω_1**	**ω_2**
**3-O-Caffeoylquinic acid**	-6.135	-2.075	4.105	4.060	0.246	2.075	3.135
**5-O-Caffeoylquinic acid**	-6.133	-2.013	4.073	4.120	0.243	2.013	2.997

**Caffeic acid**	-6.141	-2.012	4.077	4.129	0.242	2.012	2.992
**Dihydrokaempferol**	-6.376	-2.040	4.208	4.336	0.231	2.042	3.000
**Isorhamnetin**	-5.730	-2.116	3.923	3.614	0.277	2.129	3.355
**Kaempferol**	-5.948	-1.960	3.954	3.988	0.251	1.960	2.923
**Kaempferol 3-O-glucoside**	-6.244	-2.119	4.182	4.125	0.242	2.119	3.208
**p-Coumaric acid**	-6.265	-2.017	4.141	4.248	0.235	2.018	2.975
**Quercetin**	-5.961	-2.007	3.984	3.954	0.253	2.007	3.026
**Quercetin 3-O-glucoside**	-6.244	-2.099	4.172	4.145	0.241	2.099	3.162
**Quercetin 3-O-rhamnoside**	-5.869	-1.885	3.877	3.984	0.251	1.886	2.777
**Rutin**	-6.019	-1.881	3.950	4.138	0.242	1.885	2.736

Molecule	HOMO	LUMO	ESP
**3-O-Caffeoylquinic acid**	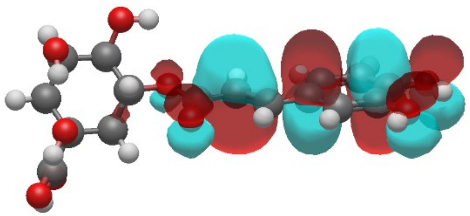	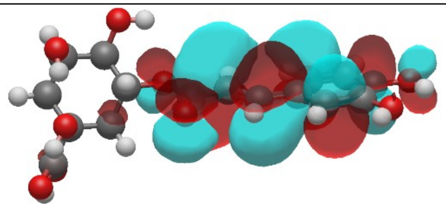	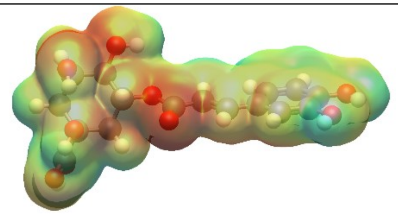

**5-O-Caffeoylquinic acid**	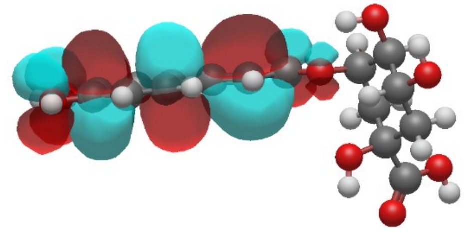	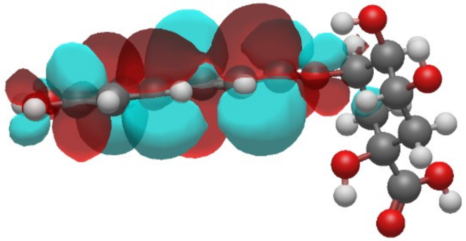	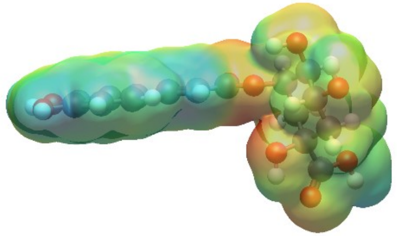
**Caffeic acid**	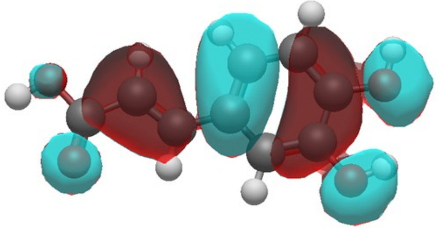	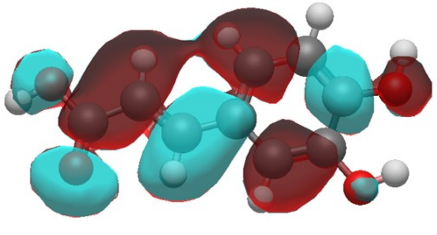	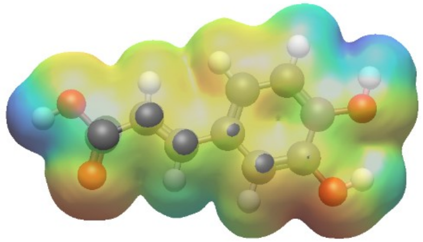
**Dihydrokaempferol**	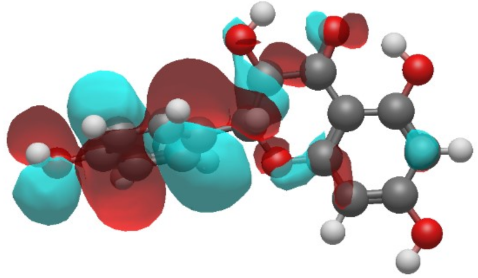	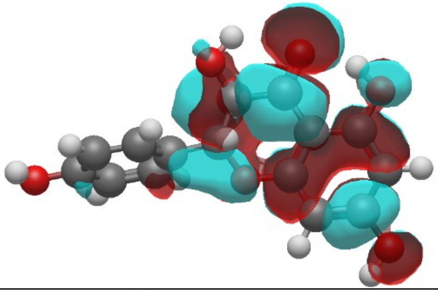	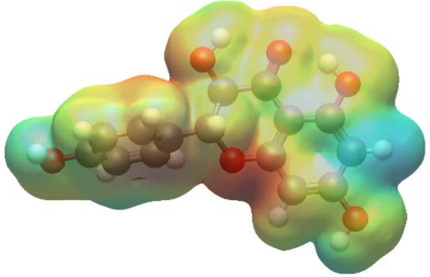
**Isorhamnetin**	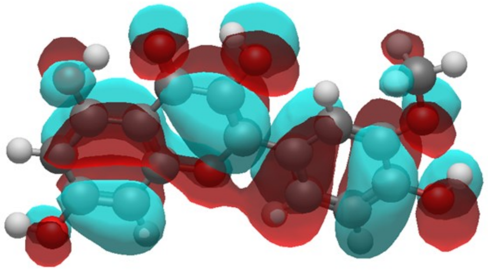	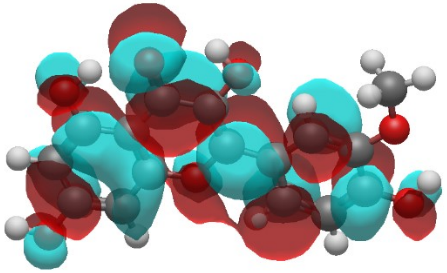	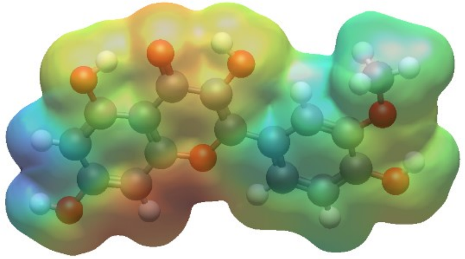

**Kaempferol**	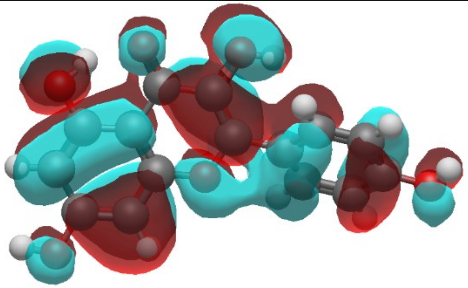	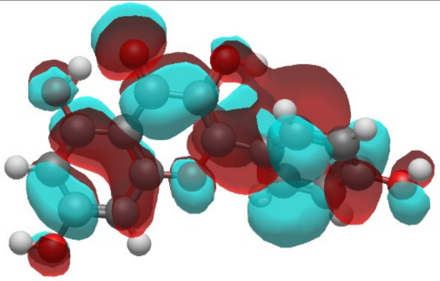	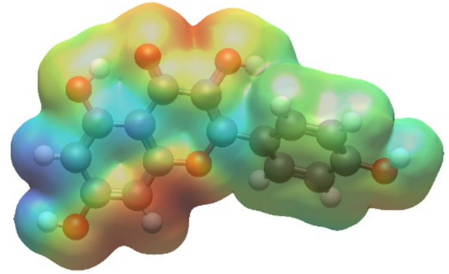
**Kaempferol 3-O-glucoside**	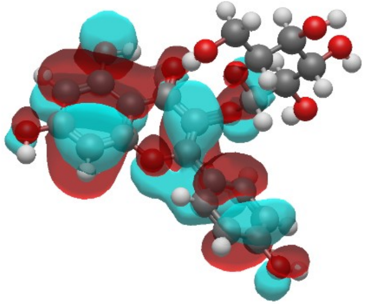	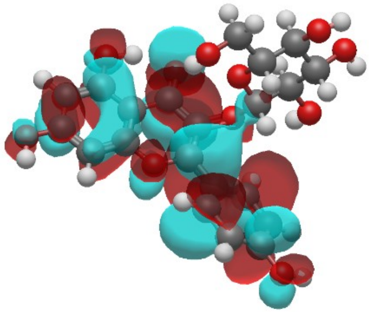	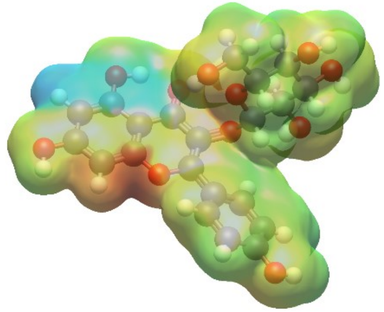
**p-Coumaric acid**	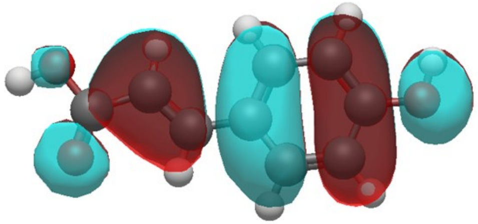	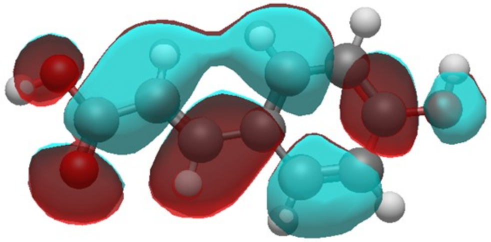	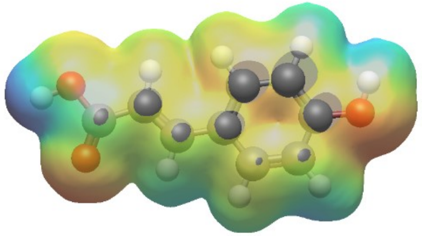
**Quercetin**	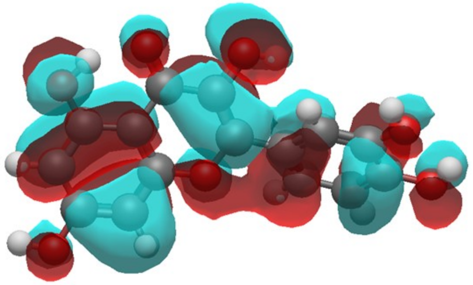	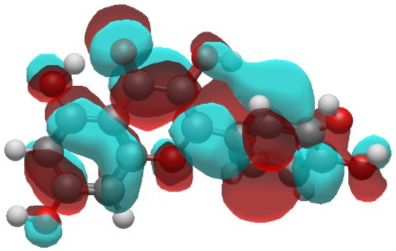	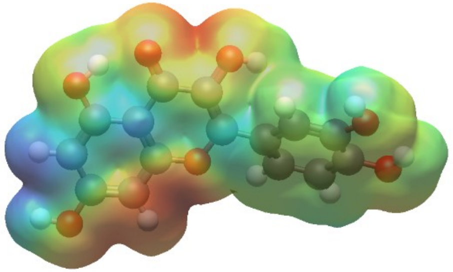

**Quercetin 3-O-glucoside**	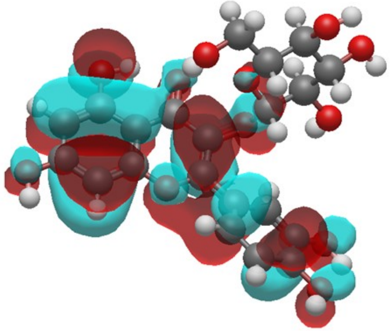	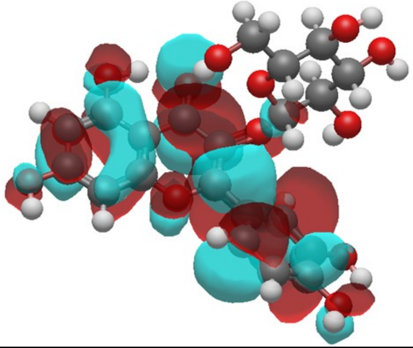	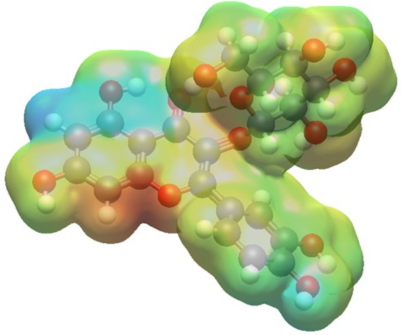
**Quercetin 3-O-rhamnoside**	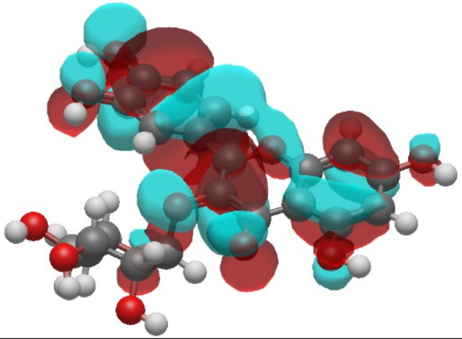	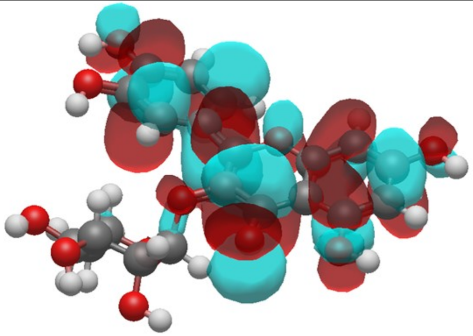	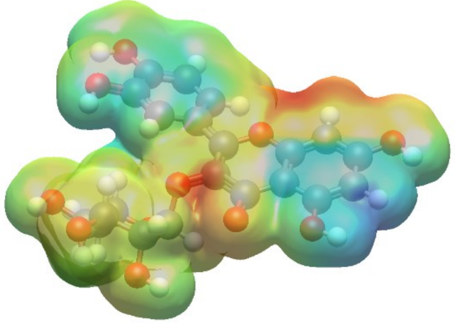
**Rutin**	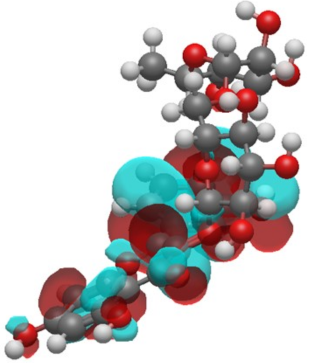	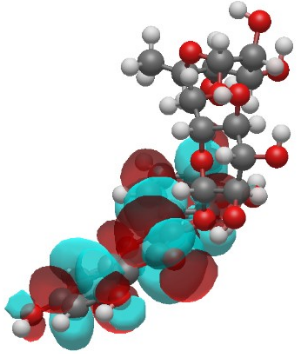	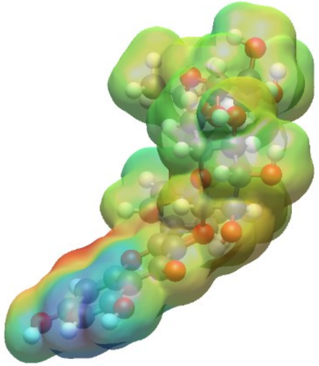

Fig. S1. HOMO, LUMO and ESP images of the dominant molecular components of the studied extracts

References

1. S. Uysal, G. Zengin, M. Locatelli, M. B. Bahadori, A. Mocan, G. Bellagamba, E. De Luca, A. Mollica and A. Aktumsek, Frontiers in pharmacology, 2017, 8, 290.
2. D. M. Grochowski, S. Uysal, A. Aktumsek, S. Granica, G. Zengin, R. Ceylan, M. Locatelli and M. Tomczyk, Phytochemistry Letters, 2017, 20, 365-372.
